# Review of Tunable Hollow Fiber Loose Nanofiltration Membranes: Fabrication, Surface Functionalization and Sustainable Water Treatment with Life Cycle Assessment

**DOI:** 10.3390/membranes16080266

**Published:** 2026-08-11

**Authors:** Jiajie Liu, Shuoqing Shi, Rui Liu, Suping Yu, Liming Dong

**Affiliations:** 1 School of Light Industry Science and Engineering, Beijing Technology and Business University, Beijing 100048, China; 2431042241@st.btbu.edu.cn (J.L.); ssq25@mails.tsinghua.edu.cn (S.S.); 2330501014@st.btbu.edu.cn (R.L.); 2 Key Laboratory of Cleaner Production and Integrated Resource Utilization of China National Light Industry, Beijing Technology and Business University, Beijing 100048, China; 3 Institute of Nuclear and New Energy Technology, Tsinghua University, Beijing 100084, China

**Keywords:** loose nanofiltration, hollow fiber membrane, membrane application, membrane preparation, life cycle assessment

## Abstract

Hollow fiber loose nanofiltration (HF-LNF) has attracted increasing attention as a pressure-driven membrane platform that combines loose nanofiltration (LNF) selectivity with the high packing density and self-supporting geometry of hollow fibers. This review critically evaluates recent advances in HF-LNF membranes, including controllable fabrication strategies, surface functionalization techniques, and practical engineering applications, with a discussion of life cycle assessment (LCA) for evaluating the environmental and economic sustainability of HF membrane systems. Phase inversion, interfacial polymerization (IP), coating, and grafting are compared in terms of structural controllability, process complexity, selective-layer stability, modification uniformity, reproducibility, and scale-up feasibility. Phase inversion is relatively compatible with continuous hollow-fiber spinning, but independent regulation of the support and selective layer remains difficult. IP provides greater control over selective-layer chemistry and effective pore size, whereas coating and grafting offer flexible surface functionalization but may be limited by additional transport resistance, layer durability, and non-uniform modification of curved surfaces. Direct HF-LNF application remains concentrated on dye/salt separation. Based on the evidence from HF-NF or flat LNF systems, the potential of HF-LNF in water softening, heavy metal removal and emerging pollutant control is analyzed. Critical challenges restricting industrial translation are discussed, including poor long-term antifouling capacity and difficulties in large-scale, low-cost manufacturing. On this basis, LCA is further introduced as a decision-support framework for identifying potential environmental hotspots in membrane manufacturing and operation, while the limited availability and comparability of HF-LNF-specific life-cycle data are explicitly recognized. Ultimately, it is proposed to focus on novel functional materials, eco-friendly preparation processes, and scaled membrane engineering, aiming to offer theoretical support for the rational design and real-world industrial deployment of next-generation HF-LNF membranes.

## 1. Introduction

Pressure-driven membrane processes span microfiltration (MF), ultrafiltration (UF), nanofiltration (NF), and reverse osmosis (RO). NF typically exhibits molecular weight cutoff (MWCO) values of 200–1000 Da and provides higher solute rejection than UF while maintaining higher water flux than RO [1,2,3]. It also exhibits moderate levels of charge due to the dissociation of functional groups on the membrane surface or the adsorption of charged solutes. For example, polymeric membranes containing carboxylic and sulfonic acid groups can develop surface charge in aqueous solutions [4] and can exhibit high rejection of small charged organic molecules. Additionally, NF membranes do not undergo phase change during operation. These combined size- and charge-based separation characteristics have supported the growing application of NF in water purification, wastewater treatment, and resource recovery.

Nevertheless, conventional NF membranes do not always provide the desired selectivity between inorganic salts and organic solutes. The relatively compact selective layers may retain multivalent ions together with organic molecules, which limits their effectiveness in applications requiring simultaneous organic-solute retention and salt passage [5]. UF membranes often allow both salts and low-molecular-weight organics to pass. To bridge this separation gap, Van der Bruggen [6] first proposed the concept of loose nanofiltration (LNF) in the context of textile wastewater treatment. A universally accepted numerical definition of LNF has not yet been established [7,8,9,10]. Reported MWCO, permeance, and salt-rejection ranges vary with solute, membrane charge, feed composition, pH, concentration, and operating conditions. Therefore, this review adopts an operational definition combining membrane structure with separation performance. Specifically, membranes with an MWCO of about 1000 Da are primarily classified as LNF membranes, provided that they can effectively retain selected organic solutes while allowing substantial passage of inorganic salts, particularly divalent or multivalent salts, under the reported test conditions. Reported numerical ranges are treated as typical characteristics rather than mandatory classification criteria.

The practical performance of LNF is also influenced by membrane geometry, hydrodynamics, and module configuration. Among the available configurations, hollow fiber (HF) membranes offer a high membrane-area-to-module-volume ratio, a self-supporting geometry, and considerable flexibility in module design [11]. Combining the selective characteristics of LNF with the HF configuration may therefore facilitate compact module construction, high-throughput treatment, and flexible operation. On this basis, HF-LNF is defined in this review as an LNF selective structure implemented in an HF configuration.

However, favorable separation performance at the membrane or module level does not necessarily guarantee an overall environmental advantage. The environmental performance of HF-LNF also depends on factors such as polymer and solvent consumption, component shell and encapsulating materials, replacement frequency, and scrap treatment during membrane spinning and selective layer preparation. Some HF-LNF designs may reduce operating energy through higher permeance or lower applied pressure, while simultaneously introducing additional environmental burdens during material production and membrane fabrication [12]. Therefore, LCA is introduced as a complementary framework for evaluating the environmental trade-offs between its manufacturing, operation, maintenance and scrapping stages.

**Figure 1:**
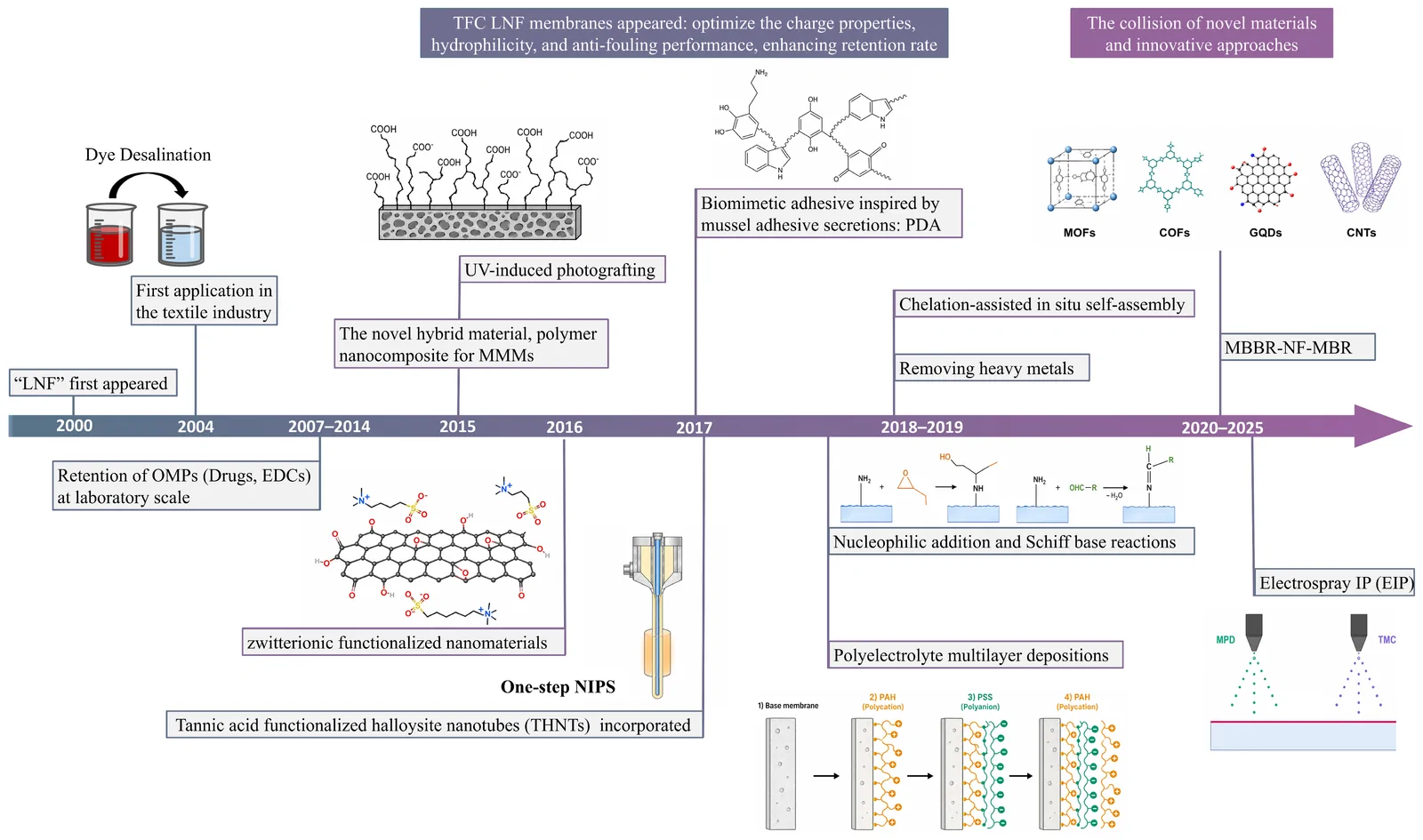
Development of LNF and HF-LNF membranes and their related applications [13,14,15,16,17,18,19,20,21,22,23,24,25,26,27,28].

A literature search was conducted in the Web of Science Core Collection, Scopus, and Google Scholar for publications available up to June 2026. The principal search terms included combinations of “loose nanofiltration”, “hollow fiber loose nanofiltration”, “hollow fiber nanofiltration” and related terms. Studies reporting HF-LNF fabrication, modification, separation performance, durability, module design, or LCA-related data were included (As shown in Figure 1). 

Studies on flat-sheet LNF, conventional HF-NF, RO, membrane bioreactors (MBR), and membrane distillation were used only as explicitly identified supporting evidence when direct HF-LNF data were unavailable. The results showed that current research has mainly focused on NF [29,30], LNF [8], and HF-NF [31,32,33,34]. Although some preliminary studies on HF-LNF membranes have been conducted, systematic reviews aimed at the emerging HF-LNF field remain limited. Accordingly, this review critically examines reported HF-LNF fabrication, functionalization, and water-treatment studies. The subsequent sections examine the design and application of HF-LNF membranes, with emphasis on their preparation methods, functionalization technologies, and potential in water treatment, with the aim of providing theoretical guidance and direction for future technological development.

## 2. Fundamentals of HF-LNF Membranes

### 2.1. Hollow Fiber Membranes and Flat-Sheet Membranes

Flat-sheet (FS) membranes are named for their planar structure and are commonly assembled into plate-and-frame or spiral-wound modules. Their accessible membrane surfaces facilitate membrane fabrication, structural characterization, module replacement, and direct physical or chemical cleaning. However, their planar surfaces may facilitate the gradual deposition of foulants and the formation of a cake layer [35]. FS modules generally require supporting plates, flow channels, or feed spacers, which occupy additional module volume. HF membranes consist of numerous self-supporting capillary fibers assembled within a compact module and can be operated in either inside-out or outside-in filtration modes. Compared with FS membranes (300~1000 m^2^/m^3^), HF membranes offer a higher packing density (750~1700 m^2^/m^3^). Their small fiber diameter and high surface-area-to-volume ratio provide a larger effective filtration area within the same module volume and thereby enhance water production efficiency [36,37]. Depending on the module design, HF membranes can also be operated using backwashing, relaxation, air scouring, or intermittent filtration to control concentration polarization and reversible fouling. Moreover, capillary geometry may improve hydraulic control and reduce reversible deposition, thereby lowering the required cleaning frequency [38,39].

### 2.2. HF-NF Membranes and HF-LNF Membranes

HF-NF and HF-LNF differ mainly in the separation characteristics of their selective layers. HF-NF membranes generally possess relatively compact selective structures and often exhibit appreciable rejection of multivalent ions together with low-molecular-weight organic solutes [40]. In contrast, HF-LNF membranes have looser structures and a wider pore size distribution, offering higher water permeance and greater salt passage while retaining target organic solutes. The relatively low salt rejection reduces the accumulation of retained salts and the associated osmotic-pressure increase when treating saline organic wastewater. These characteristics may reduce deposition and maintenance requirements, making HF-LNF membranes suitable for organic-rich wastewaters from food-processing and biomedical applications.

### 2.3. Emerging Research Directions for HF-LNF Membranes

Building on current advances in HF-LNF membranes, emerging research is mainly progressing along three interconnected directions: material innovation, structure–performance optimization, and improved understanding and regulation of separation mechanisms. These directions aim to overcome the persistent trade-offs among permeability, selectivity, fouling resistance, mechanical stability, and scalability [41,42,43]. This section provides a conceptual overview of these emerging trends and their remaining uncertainties.

#### 2.3.1. Innovation in Functional Membrane Materials

Material innovation has been investigated as a possible route for regulating HF-LNF performance. In addition to conventional polymeric and ceramic materials, emerging studies have explored nanocomposites, organic–inorganic hybrid materials, bio-based polymers, and self-healing or stimuli-responsive components [44,45,46,47,48,49,50,51]. These materials may introduce tunable pore structures, specific solute interactions, or improved mechanical and chemical stability. However, their practical value depends on compatibility with HF substrates, uniform incorporation into selective layers, resistance to leaching, and long-term stability under filtration and cleaning conditions. Therefore, most advanced material concepts should currently be regarded as emerging opportunities rather than established HF-LNF solutions.

#### 2.3.2. Structure–Performance Optimization

Membrane structure design has a decisive impact on its separation ability, flux, selectivity, antifouling properties, and long-term stability [52]. Key parameters include selective-layer thickness, pore-size distribution, surface charge, interfacial adhesion, substrate morphology, lumen diameter, and fiber-wall thickness. Reducing transport resistance can improve water permeance, but an excessively loose or non-uniform selective layer may weaken organic/salt selectivity and increase defect formation. Similarly, high packing density can reduce module footprint, whereas narrow flow channels may increase pressure loss, fouling risk, and cleaning difficulty.

Membrane configuration mainly affects lumen- and shell-side hydrodynamics, concentration polarization, and external mass transfer, whereas transport through the selective layer is governed primarily by effective pore size, membrane charge, dielectric properties, and solute–membrane interactions [53]. Straight hollow fibers do not inherently generate Dean vortices. Such secondary flows are associated with curved, helical, coiled, or woven channels and are commonly described using the Dean number, which depends on the Reynolds number and the ratio of channel diameter to curvature radius [54,55,56]. Consequently, no universal critical Reynolds number or flow-velocity threshold can be applied to all HF systems. Increasing flow velocity may instead increase pressure loss and pumping demand. Finding an optimal balance between flux and selectivity and integrating surface modification with structural optimization are key to the design of HF-LNF membranes. Future optimization should focus on balancing membrane-level separation performance with mechanical integrity, fabrication reproducibility, and module-scale hydrodynamics.

#### 2.3.3. Advances in Separation Mechanisms

**Figure 2:**
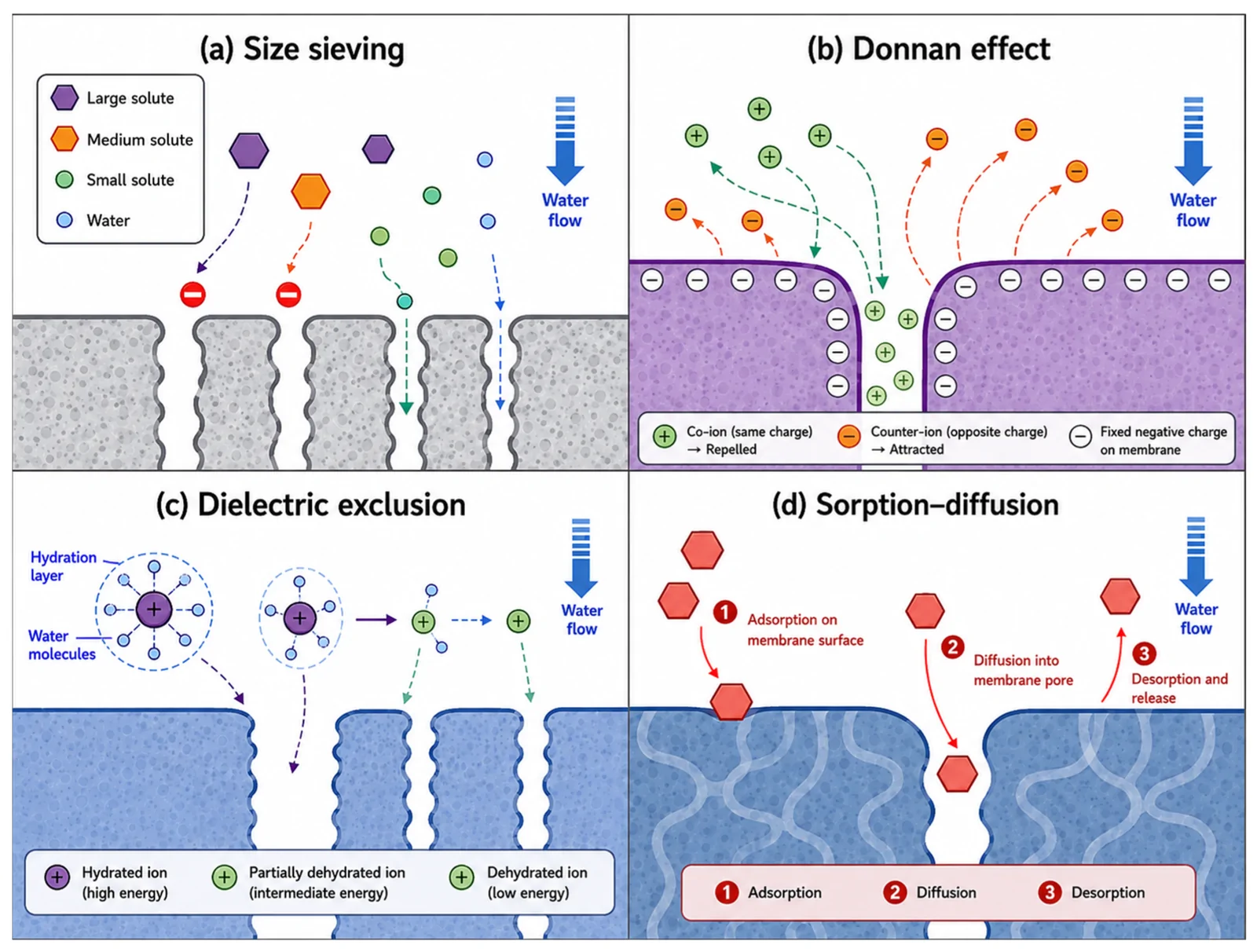
Schematic diagram of the separation mechanism of HF-LNF membranes.

Solute transport through HF-LNF membranes is governed by the coupled effects of steric hindrance, electrostatic interactions, dielectric exclusion, and hydration rather than by any single mechanism (As shown in Figure 2). Size sieving is determined by the ratio of the solute size to the effective pore size of the membrane [57]. In the separation process, small organic molecules may form aggregates through hydrophobic effects, hydrogen bonding, or van der Waals forces, thereby enhancing the rejection effect, especially when the concentration of wastewater pollutants is relatively high [58,59,60]. For ions, the relevant size is often the hydrated diameter because ions are surrounded by hydration shells in aqueous solution. When the pore size approaches or becomes smaller than the hydrated-ion diameter, deformation or partial loss of the hydration shell may introduce an additional free-energy barrier, which enhances ion rejection [61]. The Donnan effect is closely related to the charge characteristics of the membrane surface material, indicating that the interaction between charged substances and the membrane is affected by the valence and type of ions [62]. Dielectric exclusion introduces an additional energy barrier when ions enter pores with a lower dielectric constant, particularly for multivalent and strongly hydrated ions [63]. Consequently, pore size, membrane charge, ion valence, ionic strength, and hydration characteristics jointly determine solute rejection. Adsorption, swelling, and concentration polarization may further alter the effective pore size and surface charge during filtration.

The relative contribution of these mechanisms changes with membrane and feed conditions. When the effective pore size approaches the hydrated or molecular dimensions of the solute, steric sieving and hydration-related energy barriers become increasingly important [64]. For charged solutes in weakly screened solutions, Donnan partitioning may dominate, whereas increasing ionic strength compresses the electrical double layer and weakens electrostatic exclusion. Dielectric exclusion can remain relevant for strongly hydrated and multivalent ions even when membrane charge is partly screened [65,66]. For organic solutes, adsorption and aggregation may alter the apparent solute size and membrane charge, causing the measured rejection to deviate from that predicted solely from nominal molecular weight [67]. Therefore, separation data should be interpreted using the combined effects of membrane pore size, surface charge, feed ionic strength, solute speciation, hydration characteristics, and operating history.

Beyond these established effects, functional modification may introduce more specific membrane-solute interactions, including hydrogen bonding, coordination, electrostatic attraction, π-π interactions, and host–guest recognition [68]. Most supporting evidence, however, originates from related LNF or HF membrane systems rather than directly from HF-LNF membranes. At present, their stability and repeatability in HF-LNF membranes have not been fully verified, but they can provide design hypotheses for future investigation.

### 2.4. Life Cycle Assessment of HF-LNF Membranes

#### 2.4.1. Scope and Current Base

LCA is a quantitative analysis tool used to evaluate the environmental impacts of a product throughout its entire life cycle, typically covering stages such as raw material acquisition, production, use, and disposal [69,70]. It provides a structured framework for assessing energy demand, resource consumption, pollutant emissions, and other environmental burdens [71,72]. Using LCA as a tool for the design and evaluation of HF-LNF membranes can provide a theoretical basis and practical guidance for the development of more environmentally sustainable membrane technologies.

However, dedicated cradle-to-grave LCA studies specifically addressing HF-LNF membranes remain very limited (Table 1). Most currently available quantitative evidence is derived from general polymeric membrane production, conventional HF systems, MBR, membrane distillation (MD), or FS membrane configurations rather than from HF-LNF processes themselves. These studies differ substantially in functional unit, system boundary, membrane application, electricity mix, feed composition, membrane lifetime, cleaning frequency, and end-of-life assumptions. Therefore, the numerical values discussed below should be interpreted as case-specific evidence for identifying potential environmental hotspots, rather than as universally applicable environmental advantages of HF-LNF membranes.

#### 2.4.2. Representative Manufacturing and Operational Studies

HF modules generally provide a higher membrane-area packing density than flat-sheet configurations, which may reduce module volume and installation footprint [73]. Some membrane bioreactor studies reported that HF configurations required approximately 60–80% less membrane-tank volume than flat-sheet configurations at the same nominal treatment capacity. However, these results are specific to MBR module arrangements and aeration strategies and do not constitute direct environmental data for HF-LNF membranes. Moreover, higher packing density does not necessarily lead to proportional material savings because module housings, potting materials, fiber-production yield, defective fibers, and replacement frequency must also be considered [74].

Module configuration may also influence operational energy demand. In a vacuum membrane-distillation study, the HF configuration showed higher flux and gained output ratio (GOR), an improved temperature-polarization coefficient (TPC), and lower specific thermal energy consumption (STEC) than the corresponding flat-sheet system under the reported operating conditions [75], thereby further improving energy efficiency and cost-effectiveness. Nevertheless, membrane distillation is thermally driven, whereas HF-LNF is pressure driven. These results only illustrate configuration-related effects and cannot be directly transferred to HF-LNF systems.

At the manufacturing stage, polymer selection, solvent consumption, solvent recovery, and electricity supply are major environmental contributors [76]. A case-specific assessment of PVDF membrane production using NMP reported an environmental cost of approximately €766 per functional unit, with solvent-related processes contributing 40–60% of the assessed burden. Alternative ethylene carbonate (EC) or DMAc-based scenarios reduced selected environmental indicators by up to approximately 35% and 15%, respectively, while the DMAc-based scenario also reduced the reported cost by approximately €154 per functional unit [77]. The environmental benefit of replacing a conventional solvent also depends on solvent recovery, membrane performance, membrane lifetime, and the upstream production burden of the alternative solvent. If the production process itself is not environmentally friendly, such substitution could actually increase environmental costs [78].

HF geometry presents operational trade-offs. Narrow lumens may enhance wall shear and mass transfer but can also increase axial pressure loss, blockage risk, pumping demand, and cleaning frequency [79,80,81], which may affect the long-term operational stability and energy consumption of the system. One report found that the annual cleaning cost normalized by membrane area was approximately twice as high for the investigated flat-sheet system as that for the HF system (expressed as € m^−2^ membrane area yr^−1^) [82]. Nevertheless, this membrane-area-based indicator should not be interpreted as equivalent to cleaning cost per unit volume of treated water, because differences in flux, membrane lifetime, cleaning frequency, and permeate production were not necessarily normalized.

Similar limitations apply to aeration comparisons. In some MBR applications, HF systems reported air-to-water ratios of approximately 3:1–5:1, compared with 30:1 or higher for flat-sheet systems. However, the same comparison indicated that flat-sheet systems could require less blower energy per unit permeate volume [83]. Thus, conclusions depend strongly on whether aeration is expressed as airflow, membrane-area-normalized demand, or energy per unit treated water. Overall, neither configuration can be regarded as universally superior; the environmental outcome depends on the application, operating conditions, membrane lifetime, cleaning strategy, and functional unit.

#### 2.4.3. Limitations and Priorities for Future HF-LNF LCA

Current membrane LCA studies do not adequately represent the specific separation functions of HF-LNF membranes. HF-LNF is commonly designed to retain organic solutes while allowing salts to permeate; therefore, functional units based only on membrane area or treated-water volume may be insufficient. A meaningful assessment should also specify feed composition, water recovery, target organic rejection, salt passage, and permeate quality. Otherwise, a high-flux and low-pressure membrane may appear environmentally favorable even when insufficient selectivity requires recirculation, additional membrane stages, or downstream treatment. The environmental benefit of high permeability must also be evaluated together with long-term stability. Fouling, compaction, charge screening, selective-layer swelling, and chemical degradation may reduce both flux and organic/salt selectivity during operation. The resulting increases in pumping, cleaning, downtime, and membrane replacement may offset the benefits of low-pressure filtration. Therefore, lifetime-integrated production of qualified permeate is more relevant than initial pure water permeance.

Laboratory-scale membrane data also cannot be directly extrapolated to full HF modules. Although high packing density may reduce module volume, the overall impact also depends on fiber-spinning yield, defective fibers, potting materials, module housings, fiber breakage, pressure loss, flow maldistribution, and cleaning accessibility. Similarly, IP, coating, grafting, and nanomaterial incorporation increase manufacturing inputs but may reduce life-cycle impacts if they sufficiently extend membrane lifetime or reduce cleaning and energy demand. The benefit of membrane functionalization should therefore be evaluated by whether its operational savings compensate for its additional fabrication burden.

Future HF-LNF assessments should adopt a service-based cradle-to-grave framework. A suitable functional unit could be the treatment of 1 m^3^ of a defined feed to specified recovery, permeate-quality, and organic/salt separation targets. The assessment should include membrane fabrication, module assembly, pumping, cleaning, replacement, and end-of-life treatment, with sensitivity analyses for membrane lifetime, flux and selectivity decline, pressure loss, cleaning frequency, solvent recovery, and electricity mix. At present, available data are insufficient to demonstrate that HF-LNF is universally more sustainable than flat-sheet LNF or conventional NF. Its environmental advantage depends on whether low-pressure operation and compact module design can be maintained without sacrificing separation quality, durability, and module reliability.

## 3. Preparation and Modification of HF-LNF Membranes

**Figure 3:**
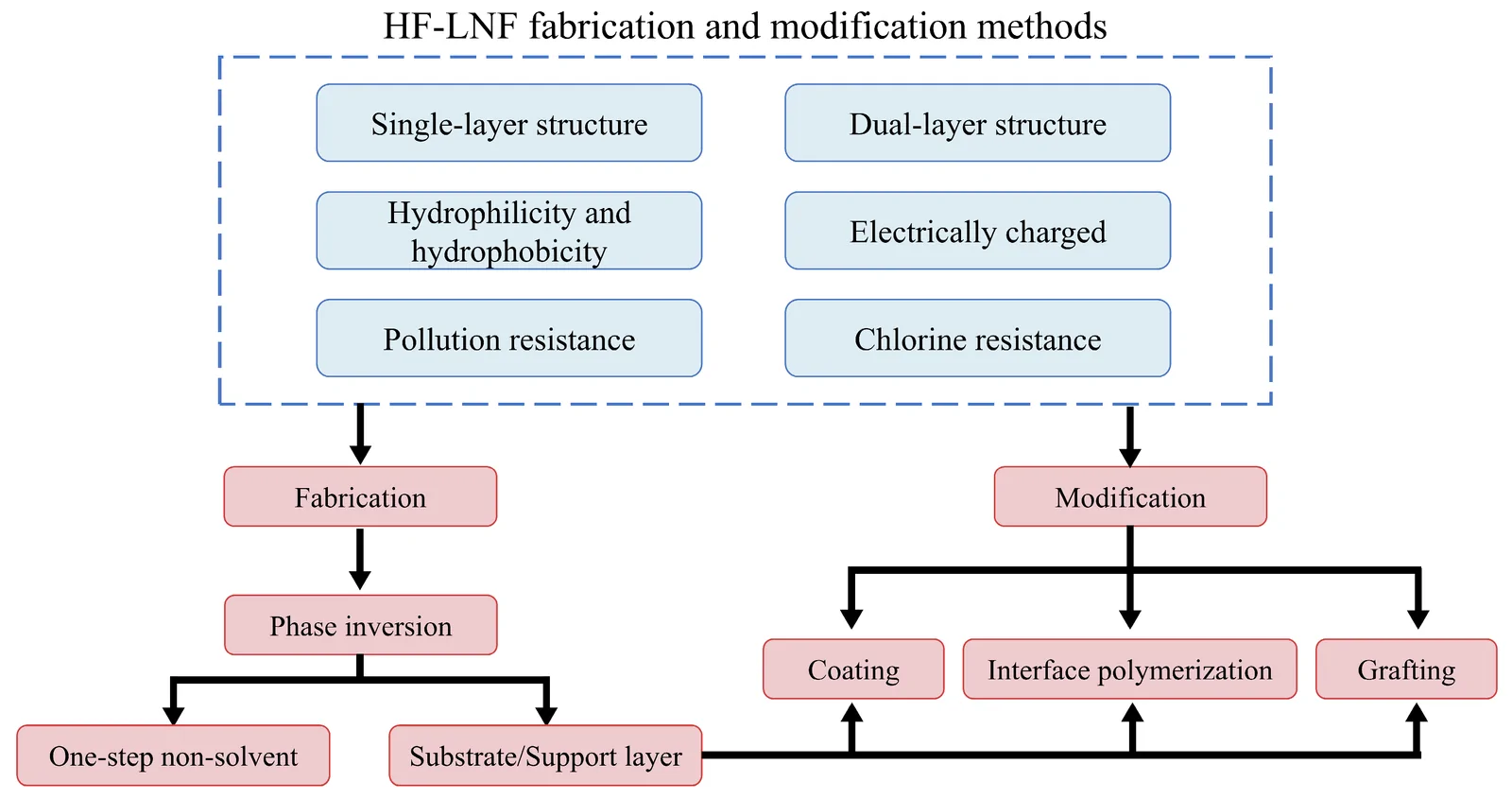
Methods of fabrication and modification of HF-LNF membranes.

Currently, reported preparation methods of HF-LNF include phase-inversion fabrication of HF substrates, in-spinning formation of selective structures, and post-fabrication surface treatment (Figure 3). HF-LNF membranes are usually divided into two categories: thin-film composite (TFC) membranes and integrally asymmetric membranes. TFC membranes are prepared by a two-step process: first, the support layer is prepared by phase inversion, and then a separation layer is formed by IP [84]. An integrally asymmetric membrane is prepared by the phase inversion method. With the continuous advancement of membrane preparation technology, controllable fabrication and surface functionalization have become key technologies for enhancing membrane performance. By introducing functional molecules or modified materials through chemical modification, coating, or grafting, the hydrophilicity, antifouling properties, and selectivity of the membrane can be optimized [85]. This section examines how these controllable preparation methods and surface functionalization technologies provide theoretical support for the design and application of HF-LNF membranes and discusses their widespread application in future water treatment technologies. However, since there are few studies on HF-LNF, we inevitably use the reports of HF-NF, LNF, or other related membrane systems as supporting evidence.

### 3.1. Phase Inversion

Phase inversion technology is a mature membrane fabrication process. Through controlled precipitation of a polymer solution, a homogeneous liquid phase is converted into a porous solid membrane [31]. The phase inversion method is mainly divided into non-solvent-induced phase separation (NIPS) and thermally induced phase separation (TIPS) [86].

#### 3.1.1. Fabrication of the Substrate for HF Membranes

Regulating phase-separation dynamics can tailor substrate porosity, surface pore size, and wettability, thereby providing a suitable support for subsequent IP or surface modification. Among many polymer materials, polyvinylidene fluoride (PVDF) is widely used because of its semi-crystalline characteristics, excellent mechanical strength, chemical resistance, and thermal stability [87]. Wang et al. [88] constructed a separation layer on a porous PVDF substrate to produce an HF-LNF membrane. The optimal membrane exhibits an MWCO of 1530 Da, showing significant differences in salt rejection rates (MgCl_2_: 0.0%, NaCl: 3.2%, MgSO_4_: 4.7%, Na_2_SO_4_: 24.5%) and dye removal rates (EBT and RB: 99.9%, MB: 96.5%), achieving effective separation between divalent salts and small organic molecules. However, the pure water permeance (PWP) of the membrane is relatively low (14.2 LMH/bar), and its MWCO and low salt rejection are consistent with the functional characteristics of LNF membranes. This also reveals the well-known permeability–selectivity trade-off challenge between high flux and high selectivity in current LNF membrane research.

Moreover, the environmental sustainability of PVDF should not be overlooked: its low biodegradability causes environmental burdens during membrane disposal after use, and large amounts of organic solvents are required in the production process, increasing resource consumption and environmental pollution. Researchers have begun exploring natural polymer materials (such as polylactic acid, gelatin, etc.) and biodegradable polymers to replace PVDF, which can not only reduce environmental burden but also improve recycling rates [89]. Using these green materials will help improve the overall sustainability of the membrane, reducing its negative environmental impact during the manufacturing and disposal stages. In addition to the replacement of the materials themselves, the optimization of the preparation process is equally crucial for reducing the full life cycle environmental impact of HF-LNF membranes. As shown in Table 2, the morphology and properties of HF membranes depend on many parameters. These parameters jointly determine surface pore size, macrovoid formation, wall thickness, mechanical strength, and surface wettability, all of which affect the subsequent formation and adhesion of an IP, coated, or grafted selective layer. Therefore, complete reporting of spinning conditions and their relationships with substrate morphology and selective-layer performance is essential for improving reproducibility and scale-up.

#### 3.1.2. Single-Layer HF-LNF Membranes

Blending modification forms a blended system with specific structures and properties by physically mixing different polymer materials, thereby compensating for the performance deficiencies of single-polymer materials [95]. Commonly used polymer materials include amphiphilic copolymers and nanoparticles [96,97]. The hydrophilic chains in amphiphilic copolymers can enhance the hydrophilicity of the membrane surface and improve the antifouling ability of the membrane, while the hydrophobic chains help to maintain the stability and selectivity of the membrane. Nanoparticles such as titanium dioxide (TiO_2_) and graphene oxide (GO), due to their high specific surface area and unique physicochemical properties, can fill the pores of the polymer substrate, improve the mechanical strength and thermal stability of the membrane, and at the same time endow the membrane with special functions such as photocatalysis [98].

In this review, one-step NIPS with in-dope modifiers is treated as an integrated fabrication–modification strategy for preparing HF-LNF membranes. This method directly incorporates the modifier into the casting solution, thereby producing integral asymmetric membranes with a single-layer (SL) structure. Unlike conventional methods that rely on post-treatment, the simultaneous fabrication and modification of the membrane reduce the risk of interfacial defects, simplify the spinning process and may improve production efficiency. It demonstrates significant advantages in rapid membrane preparation and environmental friendliness and shows potential for scalable fabrication.

**Figure 4:**
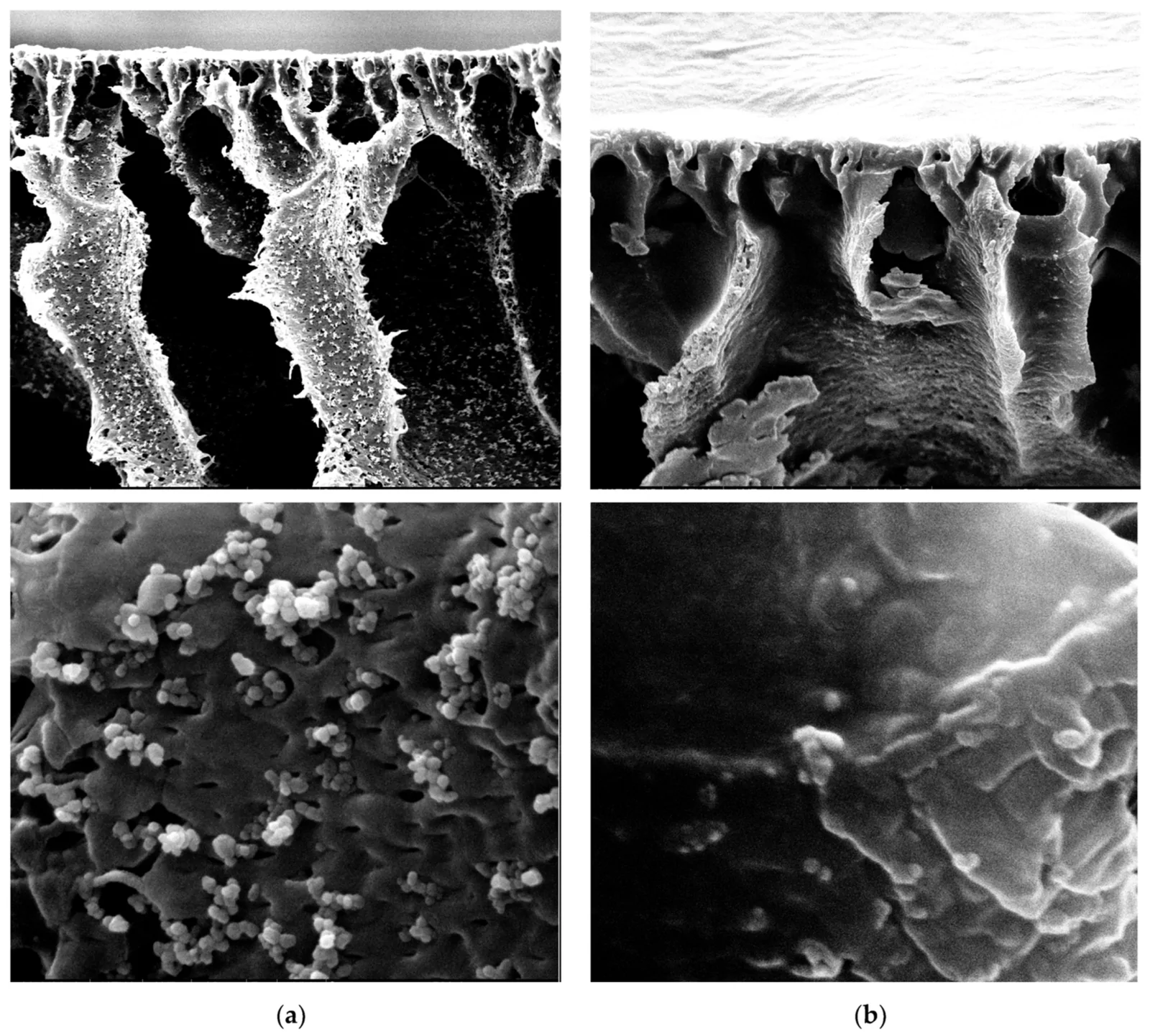
Agglomerates in the cross-section of PVDF nanocomposite membranes prepared with 50% *w*/*w* (PVDF) nanoparticles. (**a**) Reference with unmodified ZnO and 1% PVP; (**b**) only PVP-modified ZnO used. (Reprinted from [101], © 2020 by the authors. Licensee: MDPI, Basel, Switzerland).

Ji et al. [99] fabricated an HF-LNF membrane using a polyethersulfone (PES) casting solution containing 0.16 wt.% GO, with air serving as the bore fluid. The synergistic effect of air and GO inhibited the disordered stacking of polymer chains during the phase inversion process, resulting in a distinctive dense–loose bilayer structure. The carboxyl (-COOH) and hydroxyl (-OH) groups on the GO surface enhanced the membrane’s negative charge and hydrophilicity, while the nanosheet structure inhibited the formation of large pores, thereby improving its mechanical strength and permeance. At 0.2 MPa, the optimized membrane achieved rejection rates of 99.9% and 99.8% for the negatively charged dyes Congo Red (CR) and Acid Orange 10 (AO10), respectively, while maintaining a NaCl rejection below 5%. The PWP ranged from 73.7 to 95.4 LMH/bar, demonstrating relatively ideal performance. Gao et al. [100] incorporated a small amount of sulfonated polysulfone (SPSf) into the spinning dope and used PEG and PVP as hydrophilic pore-forming solutions. This approach reduced the membrane pore size and suppressed the formation of finger-like macropores. The resulting membrane achieved 96.6% rejection of indigo and maintained excellent stability and decolorization efficiency during 76 h of continuous operation, indicating its potential for textile wastewater treatment. These studies demonstrate that both the type and dosage of modifiers strongly influence membrane pore size. When the loading of amphiphilic copolymers exceeds a certain proportion, the interaction between polymer chain segments is enhanced, which may delay phase inversion and increase the membrane pore size [96]. Similarly, poor dispersion or aggregation of nanoparticles can alter the membrane pore structure (Figure 4).

However, rapid demixing may produce a relatively broad pore-size distribution or insufficiently selective pores, which can limit the rejection of low-molecular-weight organic solutes [102]. In addition, unlike TFC membranes with the porous support and a selective layer that can be optimized separately, homogeneous membranes prepared by the one-step method require simultaneous control of permeability, selectivity, mechanical strength, and surface properties within a single material system. Future studies should therefore focus on regulating phase-separation kinetics through the polymer composition, additives, bore-fluid and coagulation-bath conditions, air gap, and temperature, together with reactive additives or in situ crosslinking strategies. These approaches may provide more precise control over pore-size distribution, surface charge, antifouling behavior, and separation stability during the treatment of complex organic wastewater and thereby enhance resource recovery [103].

#### 3.1.3. Dual-Layer HF-LNF Membranes

Dual-layer (DL) hollow-fiber membranes can be fabricated by simultaneously extruding two polymer solutions through a tri-orifice spinneret (TOS), allowing the selective outer layer and porous inner support to be formed in a single spinning process (i.e., co-extrusion method) [104,105]. This co-extrusion strategy enables the composition and structure of the two layers to be adjusted independently, thereby combining surface separation functionality with mechanical support. During phase inversion, interactions between the two dopes and differences in solvent–non-solvent exchange kinetics determine the morphology and integrity of the resulting interface.

Zhang et al. [106] combined dual-layer co-extrusion with chemical crosslinking to prepare positively charged HF-LNF membranes. An amphiphilic copolymer poly(styrene-alt-maleic anhydride) (PSMA) layer was co-extruded onto a PVDF HF support and subsequently crosslinked with PEI or poly(allylamine hydrochloride) (PAH). The resulting membrane exhibited dye rejection above 99% and salt rejection below 8.5%, together with stable long-term operation and good resistance to fouling by serum albumin (BSA) and lysozyme (LYZ). This study demonstrates that a major advantage of the dual-layer configuration is the independent tailoring of the selective layer and the support [107]. In addition, localizing expensive or functional materials within a thin outer layer may reduce their overall consumption compared with single-material membranes.

Interfacial integrity in dual-layer hollow fibers depends on thermodynamic compatibility and matched NIPS kinetics between the inner and outer dopes [108]. Sufficient chain interpenetration before complete precipitation can improve interfacial adhesion, whereas large differences in viscosity, solvent affinity, or precipitation rate may cause interfacial microvoids, delamination, differential shrinkage, or excessive layer intermixing. If the outer layer precipitates too rapidly, limited chain interpenetration and differential shrinkage may generate interfacial voids or delamination. For example, the polarity difference between PVDF and PSMA may promote interfacial microcrack formation. Conversely, excessively delayed precipitation may cause excessive mixing, penetration of the outer dope into the support, or loss of a distinct selective layer. These defects can be mitigated by matching the two dope systems and controlling the dope-flow ratio, bore-fluid composition, air gap, coagulation conditions, and, where appropriate, post-crosslinking [28,109]. Therefore, future research should further optimize the balance among interfacial adhesion, selectivity, permeability, and mechanical stability.

### 3.2. Interfacial Polymerization

IP is a commonly used process for manufacturing TFC membranes [110]. This process involves two highly reactive monomers undergoing irreversible polymerization at the interface between the organic phase and the aqueous phase, with each monomer dissolved in a mutually immiscible solvent, ultimately forming a polyamide (PA) layer [111]. In the IP process, the nucleophilic monomer in the aqueous phase (mainly piperazine (PIP) and m-phenylenediamine (MPD)) diffuses into the organic phase, while the electrophilic monomer in the organic phase (mainly trimesoyl chloride (TMC)) has limited solubility in the aqueous phase [112,113]. Therefore, the two rapidly react with each other at the water–oil interface to form polymer clusters or aggregates. Because the reaction rate exceeds the diffusion rate, the rapidly formed PA layer hinders further transport of the aqueous monomer and limits continued layer growth [114]. By optimizing the reaction parameters of the IP process and introducing modifying materials, the flux and salt rejection can be tuned. Common optimization methods include adjusting the type and concentration of monomers, membrane surface modification, and post-treatment processes [115,116,117,118,119,120,121,122,123,124].

#### 3.2.1. Effect of Reaction Monomer Type on IP

Table 3 summarizes several reactive monomers that have been frequently used in IP reactions in recent years, mainly including cases where MPD or PIP is used as amine monomers to react with TMC [116,117,118,119,120]. It is worth noting that the classification of membrane types in the table (such as LNF, NF, HF-NF) is based on the performance of the membranes. For example, whether a membrane is classified as LNF mainly depends on its MWCO, its low rejection of divalent salts, and its high selectivity for small organic molecules.

#### 3.2.2. Effect of Reaction Monomer Concentration on IP

Studies have shown that the monomer concentration used in IP directly affects membrane thickness, separation efficiency, stability, and reaction kinetics, among other characteristics [136]. In reported interfacial-polymerization-based loose nanofiltration membranes, aqueous-phase monomer concentrations are commonly in the range of 0.5–3.0 wt.%, whereas organic-phase acyl chloride concentrations, particularly TMC, are generally maintained at 0.05–0.20 wt.%. Higher aqueous-phase concentrations of up to 10.0 wt.% have occasionally been employed for less-reactive polyhydroxy monomers. Due to the considerable differences in membrane-forming conditions in current research, it is difficult to systematically compare the general effects of monomer concentration on membrane performance. Therefore, the following section will focus on typical cases to analyze the effects of concentration under specific laboratory conditions.

Wei et al. [137] analyzed the influence of MPD and TMC concentration changes on membrane separation efficiency and permeation characteristics. In the MPD series, the TMC concentration was fixed at 0.5 wt./vol.%; in the TMC series, the MPD concentration was kept constant at 1.0 wt.%. The results showed that increasing TMC concentration or decreasing MPD concentration can improve water flux but reduce rejection, and a lower MPD/TMC ratio may lead to more unreacted acyl chloride groups, reducing the crosslinking degree of the PA layer. Zhang et al. [138] introduced trimellitic anhydride chloride (TAC) and TMC as co-reactive organic monomers, enhancing membrane flux by adjusting the concentration ratio and achieving a leap in performance from RO to NF. Yi et al. [139] optimized LNF membranes by adding diethanolamine (DEA) to the PIP aqueous solution. DEA suppressed the crosslinking reaction between PIP and TMC, limited the formation of the three-dimensional crosslinked network, and promoted the development of linear structures. Meanwhile, the hydroxyl groups in the molecules improved membrane permeance, with the PWP increasing from 41.3 to 170.5 LMH/bar. Further studies showed that increasing DEA concentration to improve rejection had a threshold effect, and excessive addition could not significantly improve performance.

By adjusting the concentration ratio of MPD and TMC, membrane permeability and selectivity can be optimized, but this can easily lead to an imbalance in the degree of cross-linking, which in turn affects membrane stability and efficiency. Similarly, the introduction of other monomers such as TAC and DEA also shows that different concentrations play a regulatory role in membrane performance, especially in enhancing membrane flux and adjusting selectivity. Therefore, how to seek a balance in monomer concentration adjustment to take both permeability and rejection performance into account is one of the issues that needs to be addressed in the field of HF-LNF membrane preparation. Future research may further explore precise control strategies for monomer concentration adjustment to achieve comprehensive optimization of membrane performance. For example, combining advanced characterization techniques with molecular dynamics simulation methods can be used to investigate how monomer concentration affects monomer diffusion, reaction kinetics, and PA-layer formation while also considering the synergistic effects of multiple factors under the reaction conditions.

#### 3.2.3. Interlayer

The physical and chemical defects of traditional porous substrates (such as insufficient porosity and poor hydrophilicity) severely restrict the diffusion of monomers within the pores, resulting in structural defects in the resulting PA layer [140]. This bottleneck has driven innovative developments in IP technology: the construction of a functional interlayer between the porous substrate and the PA layer, which not only helps improve membrane performance but also enhances the sustainability of the membrane. In recent years, with breakthroughs in substrate structure optimization and regulation of reaction kinetics, the interlayer has evolved from a simple transition layer to a regulatory system with amine storage and delayed release functions [141,142]. By precisely controlling the transport rate of amine monomers, the formation of a high-performance separation layer with suitable thickness and a dense structure is ensured.

Choi et al. [143] added polyvinyl alcohol (PVA) between membrane layers and found that the PWP increased to 48 LMH/bar. This change is attributed to the hydrogen bond network formed between the hydroxyl groups of PVA and PIP, which slows down the diffusion rate of monomers and thus suppresses the excessive growth of the PA layer. Similarly, Tan et al. [144] significantly improved membrane performance by adding PVA to regulate monomer diffusion and the resulting Turing-like morphology. Tian et al. [131] coated an ultrathin GO interlayer on the outer surface of a PSF HF, and the optimal membrane achieved a retention rate of 96.1% for a 2000 mg/L Na_2_SO_4_ aqueous solution. He et al. [145] constructed a tannic acid (TA)–MoS_2_ nanosheet-embedded composite polyester membrane. This membrane exhibited a PWP of 55.8 LMH/bar, 104% higher than the control membrane, and showed excellent dye/salt selectivity. The enhanced permeance was mainly attributed to the improved hydrophilicity, water-transport channels, and the formation of a thinner polyester selective layer induced by the TA–MoS_2_ interlayer.

#### 3.2.4. Control of Reaction Conditions

**Figure 5:**
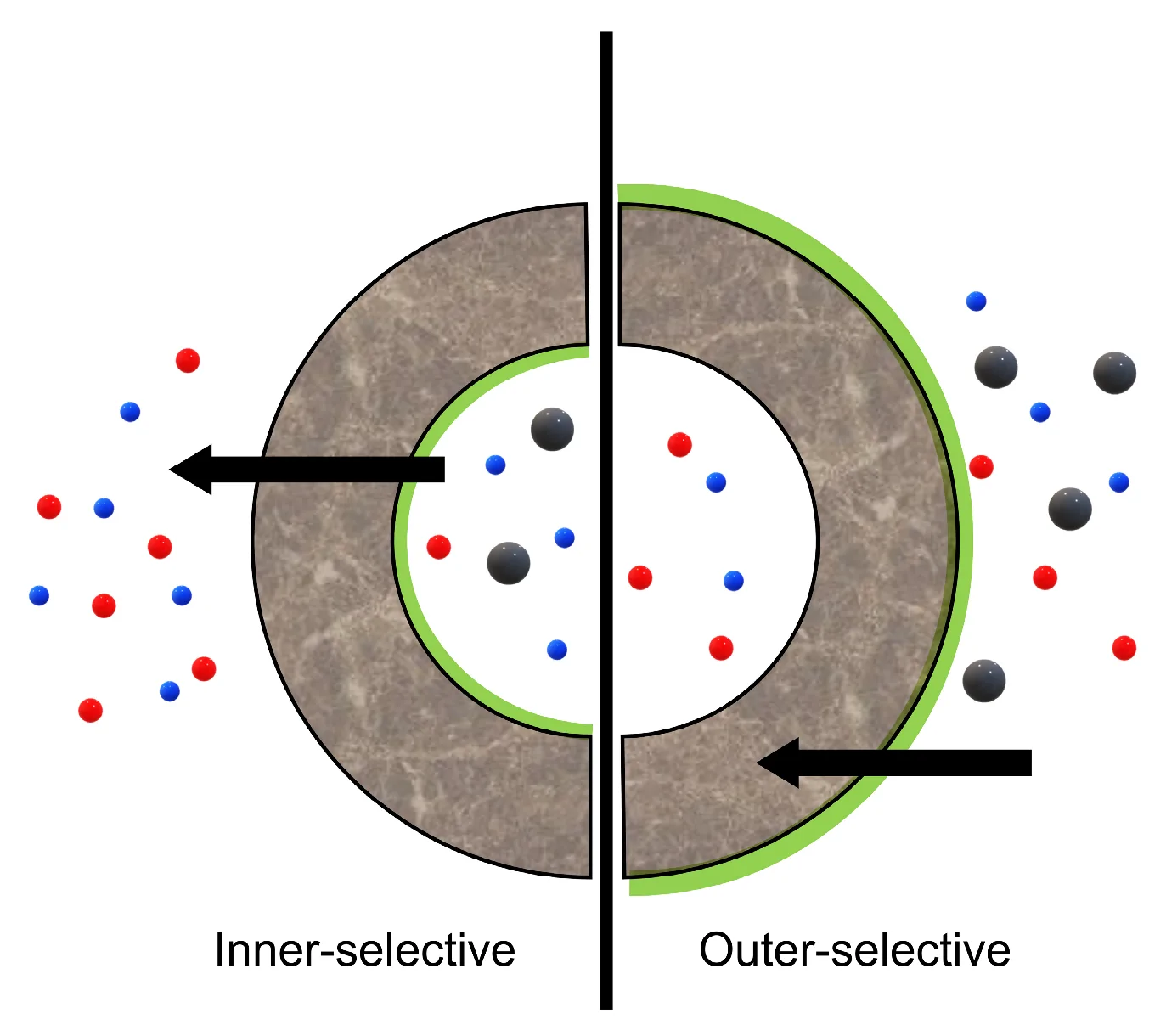
Schematic diagram of inner- and outer-selective filtration in an HF membrane.

In order to further enhance the performance and sustainability of HF-LNF membranes, researchers have extensively optimized the reaction conditions, especially in terms of reaction time and transmembrane pressure. Due to the unique structure of hollow fibers, the lumen and shell sides are physically separated, allowing IP inside or outside the lumen, and thus enabling the fabrication of HF membranes with either outer-selective or inner-selective layers (Figure 5) [146,147]. However, during in situ polymerization for outer-side selectivity, hollow fibers may come into contact with each other, resulting in an uneven PA layer that subsequently affects membrane performance. In contrast, this issue occurs relatively less frequently during the fabrication of inner-side selective membranes.

The reaction time between the aqueous phase and the organic phase is a key parameter that determines the extent of the IP reaction. Previous studies have shown that the reaction time typically ranges from 10 s to 15 min. An excessively long reaction time will lead to more aqueous-phase monomers diffusing to the newly formed active layer surface for continued reaction, resulting in increased cross-linking density of the PA layer and reduced membrane permeability. When the reaction time is extended to a certain threshold, the diffusion rate of monomers between the phases slows down, and the thickness of the PA layer gradually reaches saturation. Previous studies have referred to this phenomenon as the “self-limiting” nature of the IP process [148]. The study by Zhou et al. [149] showed that the PIP/BHTTM-TMC system reaches an optimal balance of permeance and rejection at 15 s, after which the performance tends to stabilize. Similar phenomena were also verified in the studies of Wang [150] and Dong [151]; when the reaction time exceeds the critical value (Zhou: 15 s; Wang: 60 s; Dong: 12 s), the PA layer undergoes a “self-limiting effect” due to structural densification, resulting in a decrease in flux (Dong: from 4.70 to 4.06 LMH/bar). In addition, the microscopic structure of the polymer (such as pore size, pore size distribution, and the arrangement of polymer chains) also affects membrane performance [152].

By reasonably adjusting the reaction time, the microstructure of the polymer can be precisely controlled, thereby preparing membrane materials with customized separation properties that are particularly suitable for selective separation based on specific molecular sizes. Kang et al. [19] further verified this phenomenon through electrospray interfacial polymerization (EIP) technology. By precisely controlling the electrospray time (at a rate of about 1 nm/min), nanometer-level control over the thickness, crosslinking degree, and pore size of the PA layer was achieved. The optimal membrane exhibited a high dye rejection rate (CR up to 99.6%) and a low salt rejection rate (NaCl only 6.3%). The successful application of the EIP method indicates that precise regulation of reaction time can achieve directional optimization of membrane performance, providing key technical insights for membrane fabrication under different application scenarios. When treating industrial wastewater containing high concentrations of dye molecules, appropriately prolonging the reaction time can improve the rejection rate, whereas in water treatment scenarios targeting ion removal, precisely controlling the reaction time near the critical threshold helps balance the water flux and salt rejection rate.

**Figure 6:**
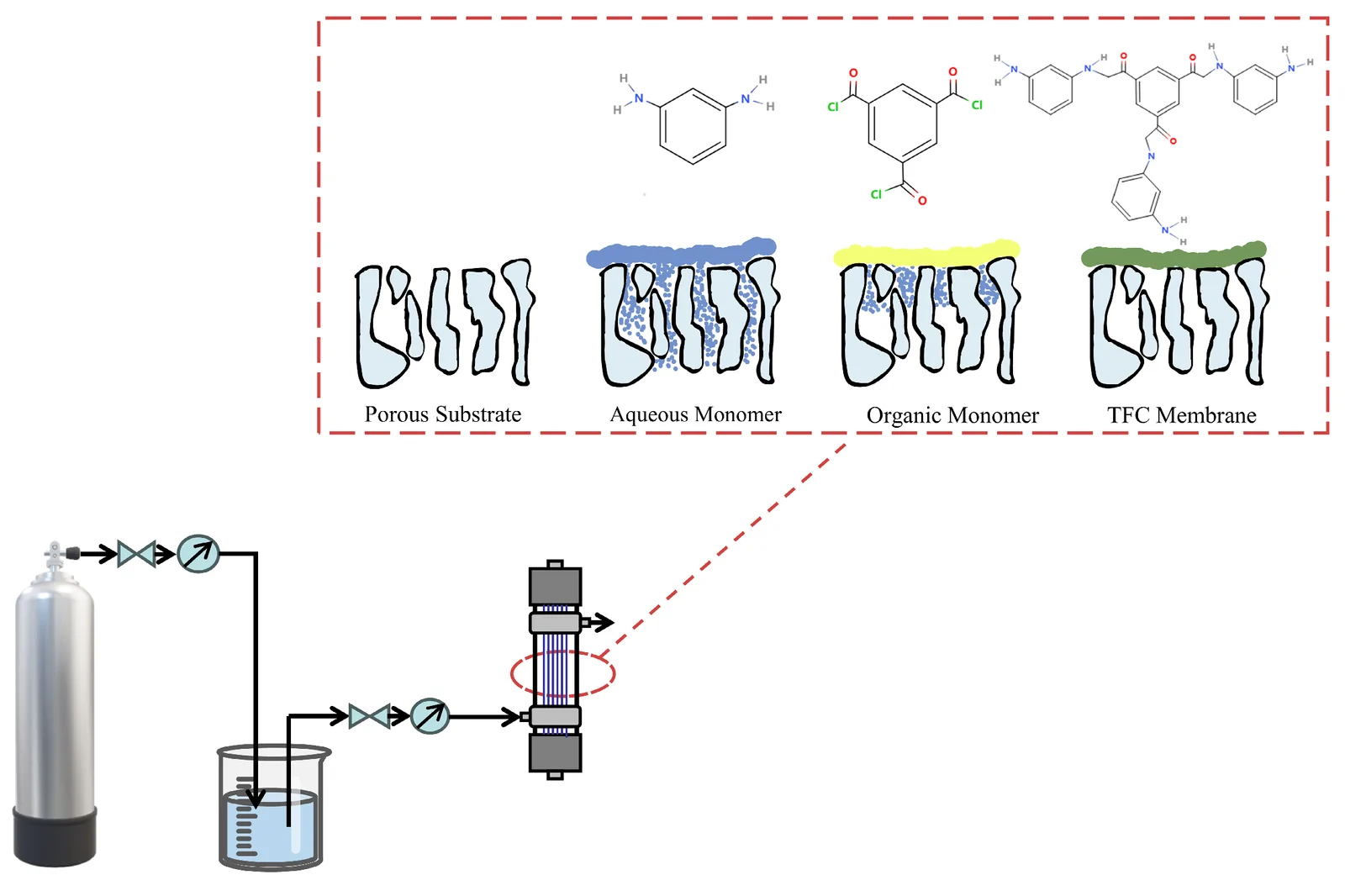
Schematic of pressure-assisted aqueous-phase circulation for HF interfacial polymerization, using MPD and TMC as examples.

Transmembrane-pressure-assisted IP has been proposed as a possible means of improving aqueous-monomer penetration and distribution within hollow-fiber substrates. Direct demonstrations in HF-LNF membranes, however, remain limited. Evidence from hollow-fiber RO and conventional NF systems indicates that a modest pressure difference across the substrate can influence monomer transport and promote the formation of a more continuous selective layer [134]. In conventional IP processes, the two phases are usually kept under atmospheric pressure. If positive pressure is applied to the water phase during the IP process while the organic phase reacts at atmospheric pressure (As shown in Figure 6), the mass transfer efficiency of the monomer will be improved, and the kinetics and mechanism of the polymerization reaction may be altered, thereby changing the structure and properties of the polymerization product. These findings provide a mechanistic hypothesis for HF-LNF fabrication but should not be interpreted as direct proof of improved HF-LNF performance. The results may be affected by the substrate pore size, wetting state, monomer concentration, and applied pressure. Dedicated HF-LNF studies are therefore required to determine whether pressure-assisted IP improves layer uniformity without increasing transport resistance or causing pore blockage.

Although the performance of the PA layer can be optimized through monomer regulation, the core challenge of adopting IP lies in controlling mass transfer at the dual interfaces. In addition, the toxic acyl chloride monomers also limit their applications in sustainable water treatment [153]. Currently, optimization strategies mainly focus on precisely controlling reaction time and reaction conditions to balance membrane flux and selectivity. Further studies can carry out physical and chemical pretreatments (such as plasma treatment) of the substrate membrane to optimize its surface properties and pore structure, laying the foundation for uniform monomer distribution and stable interface bonding [154]. Alternatively, surfactants or polymeric additives may be introduced to improve interfacial tension between the two phases, regulate monomer diffusion kinetics and reaction rate, and thus achieve fine control of the crosslinking density and microstructure of the separation layer, obtaining loose structures with high flux and high selectivity.

### 3.3. Coating

Coating technology forms a protective layer by covering the membrane surface with a functional coating, thereby regulating surface chemistry or effective pore size and improving antifouling ability, hydrophilicity, or other specific properties. The main coating methods include dip coating, spin coating, and spray coating. Dip coating and spray coating can contact curved HF surfaces more uniformly than spin coating [155]. Surface coating and polyelectrolyte self-assembly techniques can use these coating methods to treat the membrane surface.

#### 3.3.1. Surface Coating

Surface coating involves applying a solution with specific properties onto the surface of hollow fibers by means of solvent evaporation or phase transformation. IP can also be regarded as a special type of surface coating. Hydrophilic polymer coatings enhance permeability and reduce the adsorption of contaminants on the membrane surface by increasing the number of hydrophilic groups on the membrane surface [156]. Inorganic nanomaterial coatings utilize their unique physical structure and surface characteristics (such as high specific surface area and abundant pores) to provide more adsorption sites for contaminants, while simultaneously forming a physical barrier that prevents contaminants from penetrating deep into membrane pores. With technological advances, the development of new coating materials and processes is crucial for improving the performance of HF-LNF membranes. For example, nanocomposite coatings can simultaneously enhance the antibacterial activity, antifouling performance, and permeability of membranes, while the application of environmentally friendly composite materials and functionalized polymers can further optimize the stability and sustainability of membranes. These innovations not only contribute to achieving efficient and sustainable water treatment but also drive membrane technology toward a greener direction.

Abedalkader et al. [157] prepared LNF membranes with a green TA/PVA composite coating. The optimal membrane exhibited high water permeance (138 LMH/bar) and dye/salt separation selectivity, with a rejection rate for CR close to 99%, while the salt rejection rate was always below 10%. Especially notable is that the modified membrane achieved a flux recovery rate (FRR) of up to 99% for dye/salt mixtures, indicating excellent antifouling performance. Wang et al. [158] formed a PIP-monomer-rich PES coating on the outer surface of an enhanced PVDF HF porous substrate. The resulting bowl-shaped structure significantly increased the permeation surface area of the separation layer, thereby enhancing water flux.

Coating offers a relatively simple route for post-fabrication surface modification. However, the current coating process still has several limitations. Firstly, this process is easily affected by human operation and environmental factors, resulting in uneven coating thickness and concentration distribution, which in turn affects membrane performance. Secondly, most studies are still at the laboratory stage, lacking feasibility and economic evaluations for large-scale industrial production. The added layer may increase transport resistance, block pores, swell, or delaminate during filtration and cleaning. Scale-up therefore requires quantitative control of coating thickness and uniformity, together with long-term cross-flow and repeated-cleaning tests. In addition, in practical applications with high pressure or large fluctuations in operating pressure, the functional layer may collapse or rupture [159], restricting its widespread application in practical water treatment engineering.

To solve these problems, future research can explore the application of intelligent coating devices to improve the uniformity and stability of the coating. At the same time, the development of new coating materials can enable resistance to high pressure and pressure fluctuations, thereby extending the service life of the membrane. In terms of membrane sustainability, LCA is critical for assessing the environmental impact of new coating technologies. LCA can systematically evaluate the energy consumption, resource utilization efficiency and environmental impact of different coating materials and processes at various stages of the membrane life cycle, thus providing data support for future membrane design.

#### 3.3.2. Polyelectrolyte Self-Assembly

Polyelectrolyte multilayer membranes are fabricated by alternately depositing oppositely charged polyelectrolytes on a charged substrate through layer-by-layer (LbL) assembly. Through electrostatic interactions, polyelectrolyte molecules adsorb onto the substrate surface layer by layer to form a multilayer structure. By adjusting the number of deposited layers and the types of polyelectrolytes, the thickness, surface charge, and chemical properties of the separation layer can be precisely controlled, thereby optimizing membrane performance [160].

Liu et al. [18] loaded the PEI–silver ion (PEI–Ag^+^) complex onto a PAN substrate and introduced vitamin E succinate-modified silver chloride nanoparticles. Thanks to the chelation effect between PEI and metal ions, this porous structure not only effectively improves water transmission efficiency but also displays excellent dye separation performance. The removal rate for organic dyes exceeded 99%, while the rejection rates for inorganic salts (MgCl_2_, NaCl, Na_2_SO_4_) were only 6.2–12.8%. Chen et al. [161] prepared a new type of polyelectrolyte-layered composite HF-NF membrane. On a polypropylene HF substrate, CMCNa and PEI were alternately deposited, and the membrane’s structural stability was enhanced by GA crosslinking. After optimizing performance by adjusting the polyelectrolyte concentration, the MWCO of the positively charged membrane was 730 Da, showing low salt rejection (MgCl_2_ > CaCl_2_ > KCl > NaCl > MgSO_4_ > Na_2_SO_4_). This case further illustrates that for polyelectrolyte self-assembled membranes, their “loose” characteristic is reflected more in their low salt rejection caused by charge selectivity than in the pursuit of extremely high water flux.

The current polyelectrolyte self-assembly technology still faces several challenges. First, due to insufficient electrostatic forces or physical adsorption, the adhesion between the separation layer and the substrate is relatively weak [162]. This may lead to the separation layer detaching from the substrate during long-term operation, affecting the stability and service life of the membrane. Second, the multi-step deposition process of this technology is rather complicated and requires stringent operating conditions, which limits its feasibility for large-scale industrial production. In addition, the sustainability of membrane materials is also a major challenge in current research. Future studies can explore novel cross-linking methods or materials to enhance the adhesion between the polyelectrolyte layer and the substrate. In addition, considering the membrane’s LCA, researchers can optimize material selection and preparation processes, reduce the use of harmful chemicals, and improve the recycling rate of the membrane, thereby promoting the sustainable development of membrane technology.

### 3.4. Grafting

Grafting technology is a surface modification method in which functional groups or functional monomers with specific properties are grafted onto the polymer main chain of the membrane material on the membrane surface through chemical or physical methods such as irradiation or electron beams, thereby endowing the membrane surface with new functions [163]. Compared with the coating method, grafted functional layers are covalently or strongly anchored to the substrate and may provide stronger interfacial bonding. However, the uniformity of grafting is difficult to control, especially during the preparation of HF-LNF membranes. Due to the complex geometric structure of HF membranes, the grafting process is easily affected by the surface morphology and physical conditions of the membrane, which may lead to inconsistent surface properties of the membrane and thus affect the overall separation performance of the membrane. Existing grafting methods, such as electron beam [164,165], plasma [165,166] and ultraviolet/light irradiation [167,168], have been widely adopted. However, these methods often modify mainly the accessible shell surface and may not uniformly functionalize both surfaces. Therefore, research on grafting modification of HF-LNF membranes is still relatively scarce, and its feasibility for large-scale applications needs further verification.

Currently, the application of chemical crosslinking technology in HF-LNF membranes has demonstrated significant prospects. By introducing hydrophilic groups such as hydroxyl and carboxyl groups into the membrane material through chemical crosslinking, the hydrophilicity of the membrane can be enhanced, and the interaction between the membrane surface and solutes can be reduced, thereby decreasing membrane fouling. Yang et al. [24] prepared two types of HF-LNF membranes by secondary crosslinking on PVDF/SMA ultrafiltration substrates. The linear polymer poly(vinyl alcohol amine) (PVAM), which contains a large number of primary amine groups (-NH_2_) and a small number of formamide groups (-NH-CHO), was chosen as the main crosslinking agent. Through nucleophilic addition and Schiff base reactions, a dense PA layer was formed on the membrane surface. Unreacted amine groups were subsequently subjected to secondary crosslinking with GA and terephthalaldehyde (TPA), resulting in two crosslinked membranes with different structures and distinct separation layer architectures. It is noteworthy that the hybrid membrane prepared by secondary crosslinking with GA exhibited smaller pore sizes and achieved nearly 100% rejection of the low-molecular-weight dye neutral red (NR), while maintaining a PWP of 42.6 LMH/bar, demonstrating the unique advantages of crosslinked membranes in enhancing both flux and separation performance. Song et al. [169] prepared HF-LNF membranes with gradient densities from the outer to inner layers on PVDF/SMA-based membranes by reacting PEI with three different molecular weight crosslinking agents (EGDGE, PPGDGE, and TA). Based on the synergistic crosslinking effect of PEI-70k and PEI-10k, the membrane pore size was further reduced (average pore size: 0.86 nm), achieving nearly 100% removal of methyl orange (MO). The optimization of the membrane pore size enables further improvement in the separation capacity for medium- and low-molecular-weight organic compounds, while maintaining a relatively high water flux. Additionally, studies found that high-molecular-weight crosslinking agents are more suitable for tuning large-pore membranes, whereas low-molecular-weight crosslinking agents are more suitable for tuning small-pore membranes.

### 3.5. Process and Equipment Configurations for Membrane Fabrication

This section focuses on the process design of HF-LNF membrane preparation. The continuous innovation of related equipment technology provides important support for the development of HF-LNF technology. The introduction of new equipment not only improves production efficiency and reduces costs but also significantly enhances the performance and stability of the membranes, promoting the sustainable development of membrane technology.

**Figure 7:**
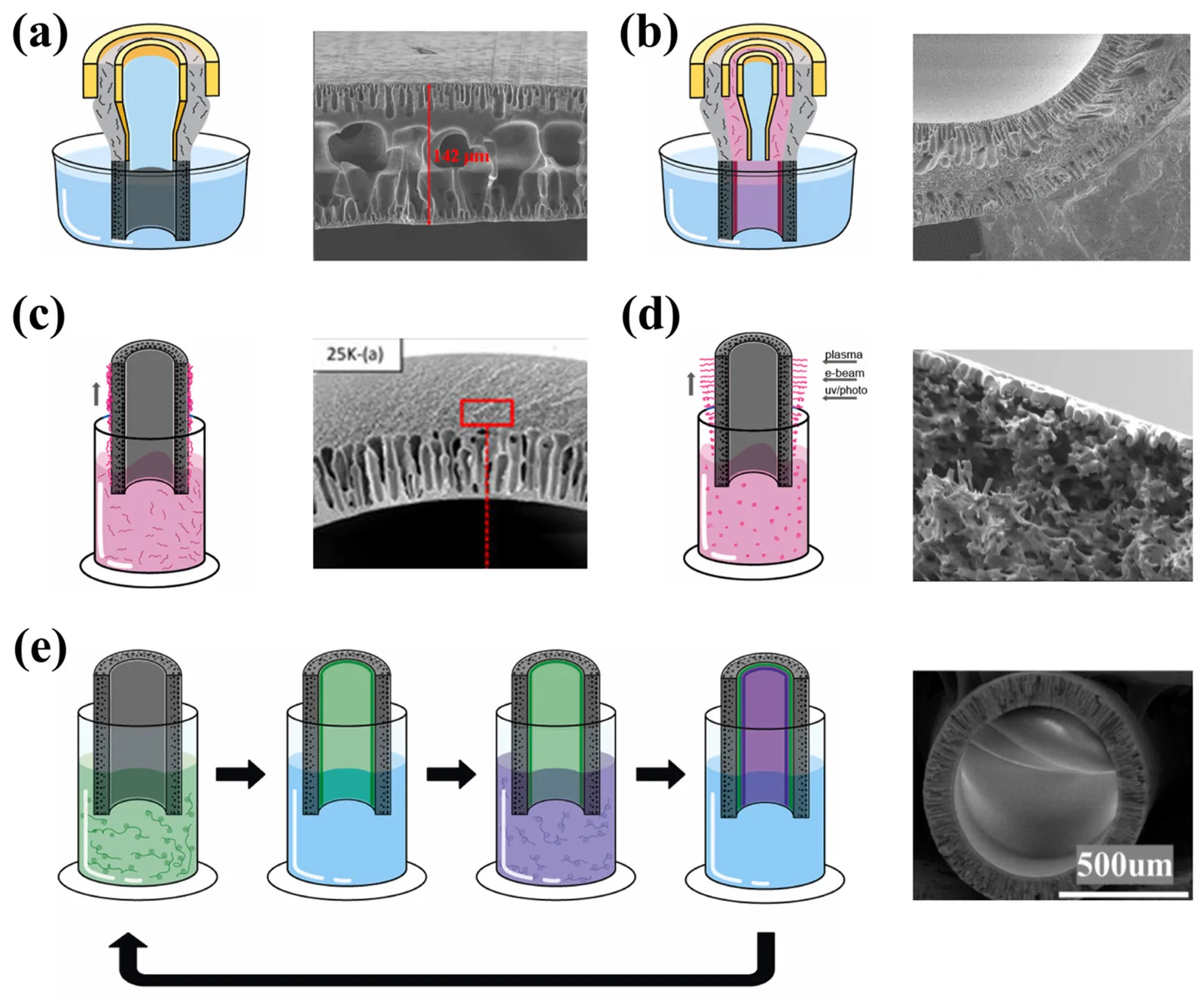
Schematic diagram of HF-LNF membrane fabrication: (**a**) single-layer membrane by phase inversion; (**b**) dual-layer membrane by phase inversion; (**c**) dip coating; (**d**) grafting; (**e**) polyelectrolyte self-assembly. (Adapted from [170] under the terms of the Creative Commons Attribution 4.0 International License; [171] © 2023 by the authors; [172] © 2023 by the authors; [173] © 2023 by the authors; [174] © 2021 by the authors; [175] © 2026 by the authors. Licensee: MDPI, Basel, Switzerland. Under the terms and conditions of the Creative Commons Attribution (CC BY) license).

For membrane formation via phase inversion processes, the membrane structure and performance can be precisely regulated by optimizing the design of the spinning spinneret. As shown in Figure 7, single-layer membranes or double-layer membranes can be prepared using a double-orifice spinneret or a TOS, respectively. The double-orifice spinneret may improve control over the thickness uniformity of single-layer membranes, ensuring the consistency of membrane separation performance, while TOS, by simultaneously extruding two different polymer solutions, can fabricate double-layer membranes with unique structures, fully leveraging the advantages of each layer to achieve optimal combinations of membrane performance. To further improve the performance of HF membranes, replacing the coagulation bath with a polymer solution for coating or grafting is also a feasible modification. The application of these new processes can effectively reduce resource consumption and environmental load during membrane fabrication and promote greener and more sustainable HF-LNF fabrication.

**Figure 8:**
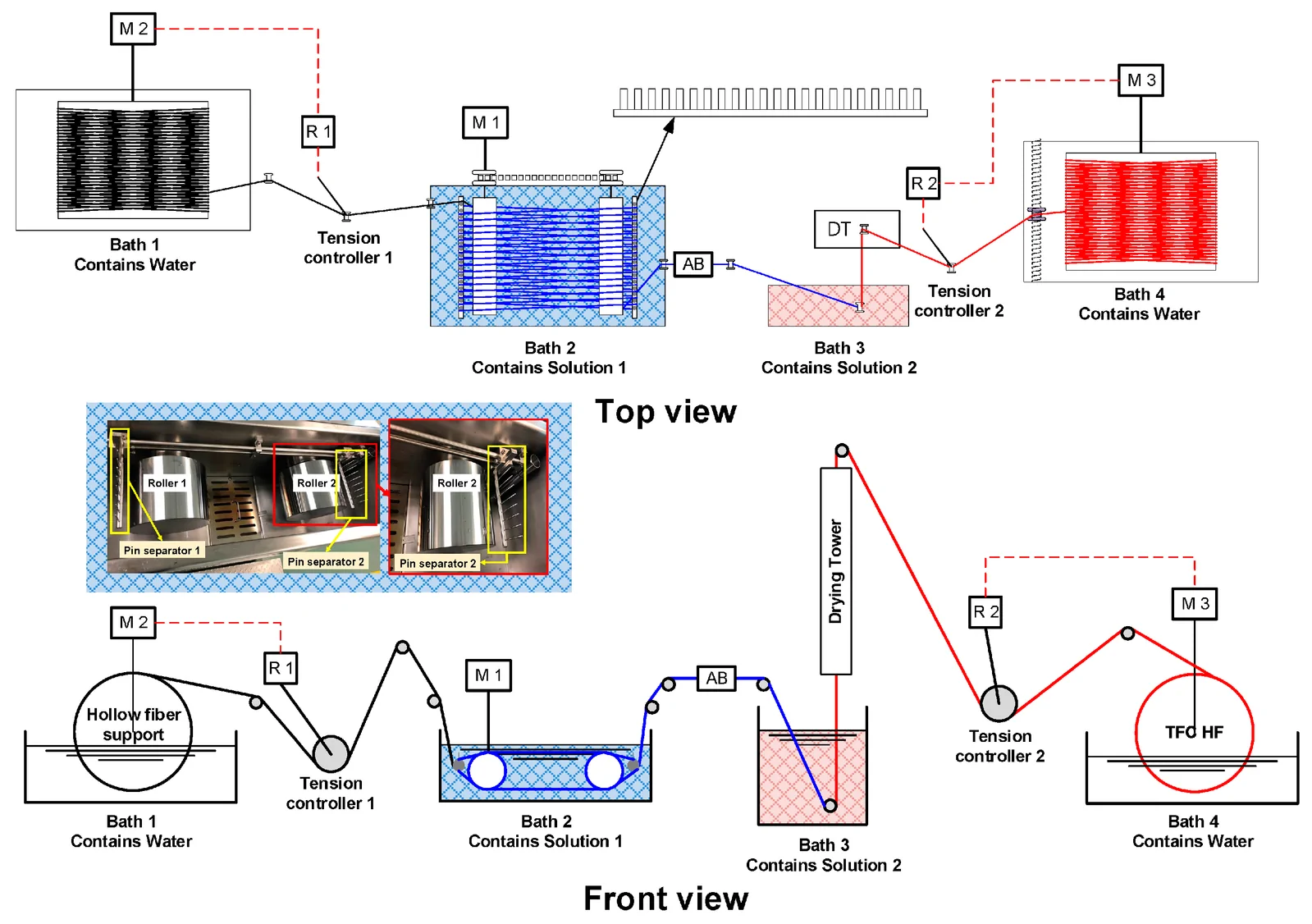
Schematic illustration of a continuous interfacial polymerization procedure for fabricating thin-film composite hollow-fiber membranes. Reprinted from [176], © 2024 by the authors. Licensee: MDPI, Basel, Switzerland.

**Figure 9:**
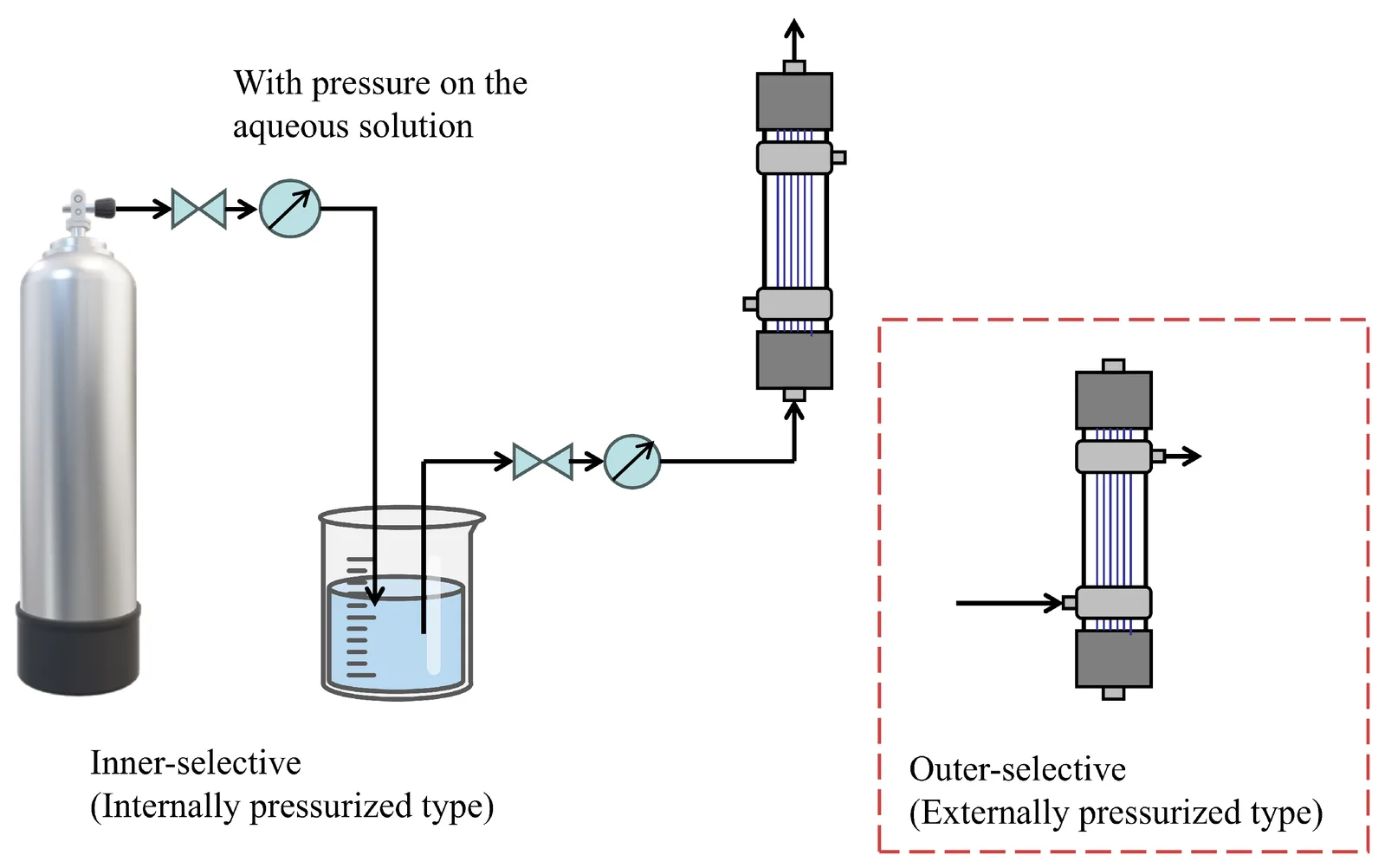
Schematic diagram of the preparation of inner- and outer-selective HF-LNF membranes by IP.

Currently, there is a wide variety of devices used for IP, with the core focus being on flexibly combining different membrane modules according to the requirements of different reaction sites. Figure 8 shows a device that achieves continuous outer-selective IP by coating modified hollow fibers. This method can effectively avoid the problem of uneven coating caused by the collision of hollow fibers with each other. Figure 9 shows a device that processes aqueous monomer solutions through pressurized circulation, which can perform treatment on the lumen side, with pressure control achieved mainly through external nitrogen. This unique advantage may improve the diffusion and reaction efficiency of the aqueous monomer within the HF. Under pressurized conditions, the aqueous monomer can penetrate deeper into the pores of the HF, reducing membrane defects caused by uneven solution concentrations and promoting the formation of a more uniform PA layer, thereby enhancing the overall quality of the membrane.

Currently, although progress has been made in the preparation of HF-LNF membranes, there are still some limitations in membrane module design. For example, the internal flow channel design of membrane modules often leads to uneven flow distribution, which in turn affects the mass transfer efficiency and overall performance of the membrane. In addition, the large-scale production of membrane modules still faces technical challenges. How to further reduce production costs and improve production efficiency while ensuring membrane performance, how to develop green and low-energy large-scale fabrication processes, and how to reduce dependence on harmful solvents and energy are important directions for future research.

Overall, module-scale fabrication depends on spinneret precision, selective-layer uniformity along the fiber length, fiber handling, potting quality, fabrication yield, and flow distribution. Continuous coating or interfacial-polymerization equipment may improve process consistency, but its benefits in energy demand, defect reduction, and production cost require experimental validation at relevant module scales. Computational fluid dynamics (CFD) can assist in identifying pressure-loss and flow-maldistribution problems, whereas the predicted improvements should be verified using realistic module dimensions, feed compositions, and long-term operation.

### 3.6. Comparative Assessment of Fabrication and Modification Strategies

The above preparation and modification strategies each have their own advantages and limitations, and in the preparation of HF-LNF membranes, no single approach can be regarded as universally superior. Phase inversion is the most direct and mature route for fabricating integrally asymmetric hollow fibers and is relatively compatible with continuous spinning and large-scale production. However, in this method, a porous support and a selective layer are formed simultaneously, making it difficult to independently optimize pore size, flux, selectivity, and mechanical strength. The resulting membrane structure is sensitive to changes in polymer concentration, solvent–nonsolvent exchange, bore fluid composition, air gap, and coagulation conditions, factors that may affect batch-to-batch reproducibility.

IP separates the preparation of the porous support from the formation of the selective layer, thus offering greater flexibility in controlling surface chemistry, charge, effective pore size, and selective layer thickness. This method is particularly attractive for achieving high organic solute/salt selectivity. Nevertheless, its application to hollow fibers faces additional challenges, including multi-step processing, uneven monomer distribution on curved surfaces, defect formation, and insufficient adhesion between the selective layer and the substrate. The coating method provides a relatively simple approach for introducing hydrophilic, charged, or antifouling materials after HF formation. However, excessive coating may increase transport resistance or block membrane pores, while physically deposited coatings may swell, peel off, or undergo performance degradation during long-term operation and cleaning. Compared with coating, grafting reactions can form stronger bonds between functional groups and the membrane substrate, potentially improving chemical and operational stability. Its main limitations lie in the difficulty of achieving uniform modification on the lumen and shell surfaces, the need for additional activation or reaction steps, and the limited evidence regarding large-scale reproducibility.

From an application perspective, when process simplicity, production efficiency, and moderate separation performance are prioritized, the phase inversion method is more suitable. IP offers greater independent control of selective-layer chemistry at the cost of additional processing and defect control. Coating and grafting are useful when post-fabrication surface tuning is required. The coating method is suitable for rapidly adjusting surface properties and enhancing antifouling performance, while the grafting method is more appropriate when strong adhesion and long-term surface stability are needed. Hybrid strategies can combine these advantages but also increase preparation complexity, chemical consumption, and quality control requirements. Therefore, the selection of the HF-LNF preparation route should be based on the target separation task, required durability, manufacturing feasibility, and environmental load, rather than solely on the highest short-term flux or rejection rate. Furthermore, since most reported membranes are evaluated under different feed compositions and operating conditions, direct comparisons remain limited.

## 4. Applications of HF-LNF Membranes

**Figure 10:**
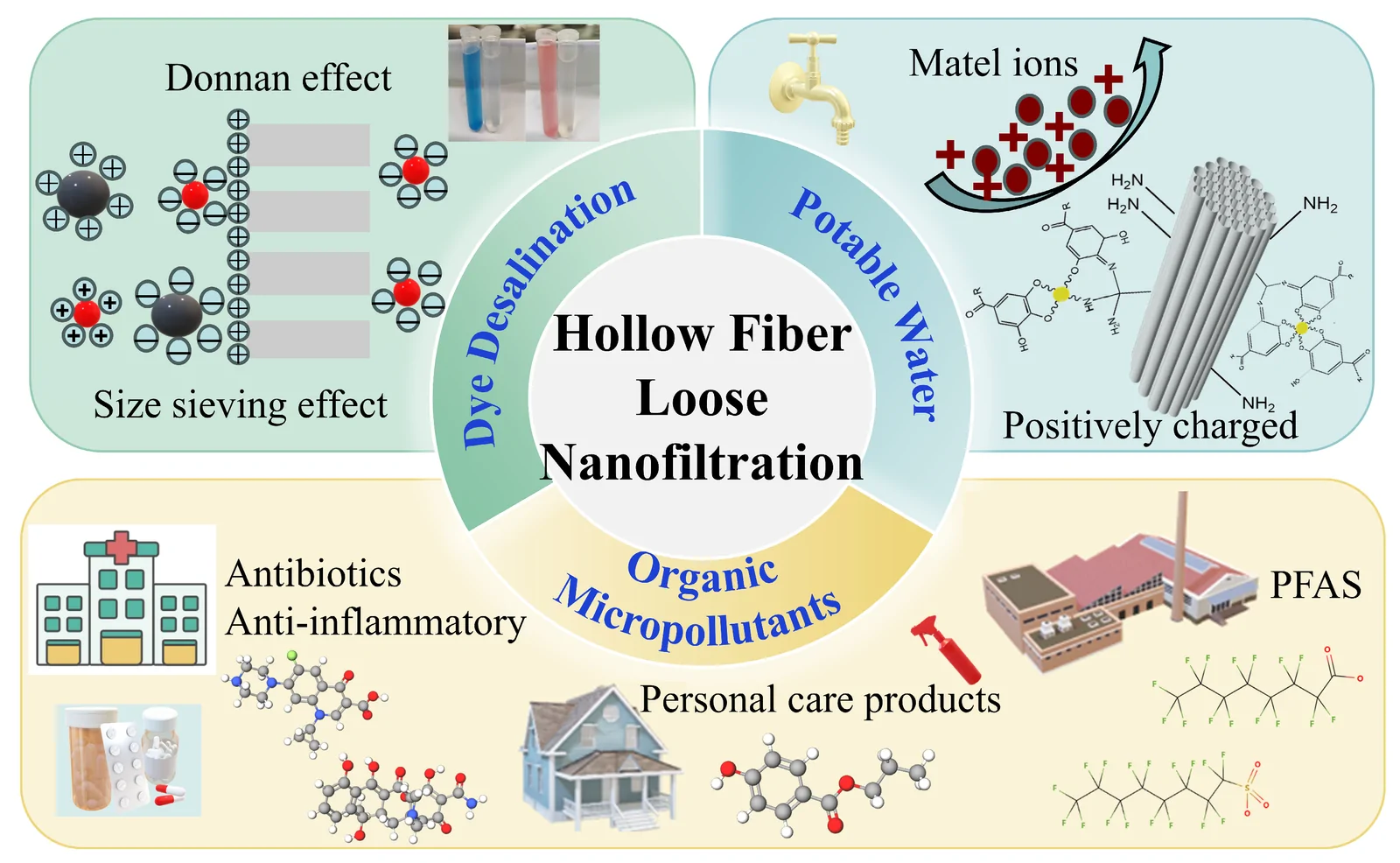
Principal application areas and separation principles of HF-LNF membranes.

In recent years, HF-LNF technology has demonstrated significant application advantages in the field of water treatment (Figure 10). This section summarizes reported applications and identifies relationships among membrane structure, performance, and operating conditions. Unfortunately, the currently available application studies directly involving HF-LNF membranes are predominantly concentrated on dye/salt separation, while their use in other water-treatment fields remains insufficiently explored. Therefore, in the following sections, evidence from flat-sheet LNF, conventional HF-NF, and other closely related membrane systems is introduced as supporting evidence to discuss the potential applicability of HF-LNF membranes in these areas.

### 4.1. Dye Desalination

Synthetic dyes are widely used in industries such as papermaking, plastics, rubber, and textiles due to their bright colors and stability [22]. With the rapid development of the printing and dyeing industries, the discharge of dye-containing wastewater has increased. Untreated effluents threaten the environment and human health. At the same time, salts such as NaCl and Na_2_SO_4_ used in dye production and dyeing processes not only increase the salinity and chemical oxygen demand (COD) of the wastewater but also further complicate the treatment of dye wastewater, especially by affecting its biodegradation process [177,178].

High dye rejection by membranes should be interpreted with caution because transient adsorption and dye aggregation may reduce the permeate concentration during the initial filtration period and thus overestimate the intrinsic membrane selectivity. The extent of adsorption depends on both dye properties and membrane-modification materials, particularly their surface charge, aromatic structure, hydrophilicity, and specific surface area. Dye molecules are usually composed of aromatic hydrocarbons and heterocyclic compounds containing chromophores and polar groups (Table 4), which confer strong chemical stability and increase the difficulty of wastewater treatment. For example, a CeZnFe-LDH-modified LNF membrane showed only a slight increase in static dye adsorption from 3.21 to 3.42 mg/g, suggesting a limited adsorption contribution under those specific experimental conditions [179]. In contrast, GO membranes exhibited much stronger adsorption of cationic methylene blue than anionic methyl orange, and the adsorbed dye altered the interlayer structure and membrane permeance [180]. Therefore, dye rejection should, where possible, be evaluated together with adsorption measurements, feed-permeate-retentate mass balance, long-term filtration after adsorption saturation, and possible dye aggregation, which may increase the effective solute size.

HF-LNF membranes demonstrate significant advantages in selective dye/salt separation. The core design principle lies in customizing the membrane’s surface charge and effective pore size according to the target dye molecules. Current studies mainly customize the separation layer of the membrane through functionalization strategies to adapt to the molecular weight and charge characteristics of different dye molecules. Bian [181] and others synthesized PVDF/SMA-based membranes using TIPS and developed three types of HF-LNF membranes with different separation layers—G-PEI, M-PEI, and C-PEI—using chemical crosslinking and metal ion coordination methods. These membranes achieved high-efficiency retention of Acid Red 27 (AR27), Sunset Yellow (SY), and Crystal Violet (CV), respectively, with retention rates of 99.5%, 99.3%, and 96.1%, while ensuring high flux. Wang [182] and others prepared LNF membranes with high dye/salt selectivity by co-depositing TA and PEI on PVDF HF substrates. The optimized membrane permeance was 32 LMH/bar, with a retention rate for CR of 99% and retention rates for NaCl and Na_2_SO_4_ of 2.3% and 6.4%, respectively.

Although HF-LNF membranes have already demonstrated technical feasibility in dye desalination, their large-scale application still faces multiple challenges. First, a permeability-selectivity trade-off remains. Second, the presence of complex wastewater components—especially surfactants and oils—will exacerbate membrane fouling and reduce flux. At the operational level, both the pH and temperature of the feed solution can affect the dissociation state of the dye and the surface charge of the membrane, thereby influencing separation performance. Therefore, in practical applications, operational conditions should be optimized according to the wastewater quality. For example, for negatively charged membranes and anionic dyes, increased deprotonation under alkaline conditions may strengthen electrostatic repulsion. Moreover, the operating pressure needs to strike a balance between flux and selectivity and should be optimized according to the specific characteristics of the wastewater and the membrane material’s tolerance. Additionally, in large-scale industrial applications, the size of the membrane module and the operating scale are factors that must be carefully considered. Especially at the pilot stage, sufficient performance verification is necessary to ensure that the membrane can maintain efficient and stable operation during scale-up.

### 4.2. Treatment of Potable Water

#### 4.2.1. Removal of Hardness Ions

Population growth and urbanization have increased pressure on drinking-water sources, including problems associated with elevated hardness [183,184]. Water hardness is mainly associated with dissolved Ca^2+^ and Mg^2+^. In drinking-water treatment, the objective may be selective hardness control rather than complete demineralization [185]. Most HF studies reporting high Ca^2+^ and Mg^2+^ rejection involve conventional HF-NF membranes rather than direct HF-LNF systems.

Zhang et al. [186] compared the performance of six commercial LNF membranes and one traditional NF membrane in treating the effluent from high-efficiency sedimentation basins of surface water, and the results showed that negatively charged LNF membranes exhibited excellent selective removal ability in advanced purification of drinking water, particularly excelling in the removal of multivalent ions. In a related study, Tang et al. [187] fabricated a positively charged HF membrane by constructing an organic-inorganic hybrid separation layer containing BaSO_4_ or CaC_2_O_4_ through PVAM-TA-metal-ion co-deposition, biomineralization, and dual cross-linking. The rejection efficiency of Ca^2+^ and Mg^2+^ was maintained above 99% at a PWP of 53.4 LMH/bar.

High Ca^2+^ and Mg^2+^ rejection can arise from different mechanisms. Positively charged membranes may reject cations through Donnan exclusion, whereas negatively charged or mixed-charge membranes may show high apparent rejection through steric restriction, dielectric and hydration effects, adsorption, complexation, or counter-ion coupling. When treating high-hardness water sources, higher concentrations of Ca^2+^/Mg^2+^ ions may cause crystallization on the membrane surface and lead to fouling, thus resulting in membrane flux decline. Therefore, future research should focus on optimizing membrane surface properties, such as dynamically regulating the membrane’s surface characteristics through the introduction of chelating groups or functional polymers, in order to achieve efficient removal of hardness ions while retaining appropriate amounts of minerals to meet the health standards for drinking water. Operating pressure, cross-flow velocity, and temperature should be optimized together with membrane structure and feed-water quality.

#### 4.2.2. Removal of Metal Ions

Heavy metal pollution has become a global environmental problem, particularly in regions where industries such as mining, electroplating, and metallurgy are rapidly developing [188]. Heavy metal ions such as lead (Pb^2+^), chromium (Cr^3+^), and cadmium (Cd^2+^) possess strong toxicity and are difficult to degrade in the natural environment. If discharged directly without proper treatment, they will cause serious harm to water quality and human health [189]. Most heavy metal ions are positively charged, and many industrial wastewaters containing dissolved metals are acidic, which makes acid resistance and membrane charge characteristics key areas of research [190]. The development of NF membranes that can effectively treat and recover heavy metal ions is of great significance for promoting environmental sustainability, protecting human health, and facilitating resource recycling [191,192]. Heavy metal rejection depends on ion valence, hydrated size, pH-dependent speciation, membrane pore size, surface charge, and background electrolyte composition. Like-charge electrostatic repulsion can enhance cation rejection by positively charged membranes, whereas negatively charged surfaces may promote cation adsorption or complexation, which does not necessarily represent stable membrane separation. Most currently reported examples originate from conventional HF-NF or flat-sheet LNF systems and therefore provide supporting design principles rather than direct evidence of full-scale HF-LNF performance.

Tang et al. [193] successfully removed metal ions such as Fe^3+^, Cu^2+^, Mn^2+^, and Zn^2+^ with HF-NF membranes developed using polymer-anchored co-deposition technology, achieving efficiency above 95% while maintaining high flux. Mohammad et al. [194] developed a positively charged hybrid LNF membrane by self-assembling ethylenediamine-grafted multiwalled carbon nanotubes (ED-g-MWCNT) on the surface of a PSF membrane. The rejection rates for Zn^2+^ and Pb^2+^ were 96.7% and 90.5%, respectively, and flux reached 80.5 LMH. Shirazi et al. [195] prepared a highly positively charged PES/CPES hollow-fiber NF membrane through hyperbranched polyethyleneimine (HPEI) self-assembly, glutaraldehyde cross-linking, and methyl iodide (MeI) quaternization. The optimized membrane achieved rejection rates of 99.8% for Zn^2+^ and 98.6% for Mg^2+^. Yin et al. [40] further enhanced the positive surface charge of a polyamide hollow-fiber NF membrane through secondary IP. The resulting membrane exhibited a PWP of 16.0 LMH/bar and rejection rates of 97.6% for MgCl_2_, 99.8% for Cu^2+^, and 99.7% for Zn^2+^.

Collectively, these studies demonstrate that positively or mixed-charge HF-NF membranes can effectively remove heavy metal ions, mainly through the combined effects of Donnan exclusion and steric hindrance. Nevertheless, most reported membranes exhibit relatively high multivalent-ion rejection and are therefore more appropriately classified as conventional HF-NF rather than HF-LNF. Research on highly permeable HF-LNF membranes specifically designed for selective heavy metal removal remains limited. Future studies should tailor membrane charge and pore size to specific metal speciation, concentration, and pH while balancing recovery, stability, and energy demand.

### 4.3. Emerging Organic Micropollutants

Emerging organic micropollutants (OMPs) have become an important environmental concern as water pollution has intensified (Table 5) [196]. These pollutants mainly include personal care products (PCPs), pharmaceutically active compounds (PhACs), and endocrine-disrupting chemicals (EDCs) [197]. EDCs, such as estrogenic compounds and pesticides, possess complex properties and multiple mechanisms of endocrine disruption and can affect the endocrine and developmental processes of mammals even at low concentrations [198,199]. PhACs mainly include drugs used to treat human and animal diseases, such as antibiotics and sedatives [200]. Due to their chemical stability, PhACs may re-enter the human body through the water cycle or food chain, thus posing potential risks to health. Several studies have reported that conventional NF membranes, especially those prepared with thin-film composite polyamide materials, have unsatisfactory retention performance for neutral hydrophobic EDCs [201,202,203].

Effective OMP removal requires tailoring the physicochemical properties of the membranes according to the molecular size, charge, hydrophobicity, and spatial structure of the OMPs. For example, for charged OMPs (such as certain antibiotics), adjusting the surface charge of the membrane can enhance electrostatic interactions; for neutral hydrophobic OMPs, it is necessary to finely tune the membrane pore size (close to or slightly smaller than the pollutant molecule size) and possibly introduce specific adsorption sites. The hollow channel design generates high shear rates through internal pressure operation and may suppress the adsorption and deposition of OMPs on the membrane surface. The high packing density enables higher OMP treatment efficiency per unit volume compared to FS membranes. In addition, the self-supporting structure of HF membranes eliminates the need for extra supporting layers, greatly reducing energy consumption. However, direct HF-LNF evidence for broad-spectrum OMP removal remains limited.

Tang et al. [204] introduced PVP onto PVDF HF substrates by TIPS, finely adjusted the surface pore size of the membrane, and then performed IP. The optimized membrane exhibited improved hydrophilicity, with the zeta potential becoming more negative (−43.96 mV), resulting in a tetracycline rejection rate of 98.9% while also improving the selectivity between Ca^2+^ and antibiotics (37.27). Sun et al. [205] synthesized a cationic composite membrane by crosslinking PEI and polyamide-imide (PAI) to treat ciprofloxacin in aqueous solutions. Experimental results showed that compared with conventional commercial IP-fabricated negatively charged membranes, this membrane exhibited superior rejection and antifouling performance. This research provides a basis for using HF-LNF membranes in the treatment of pharmaceutical-containing wastewater. In addition, Wang et al. [206] developed an HF-NF membrane using PMIA and conducted experiments to evaluate its performance in removing trace amounts of perfluorooctane sulfonate (PFOS) from water. Results indicated that, within a certain pH range, the removal efficiency of PFOS increased with higher Ca^2+^ concentrations in the influent, reaching a maximum removal rate of 99.4%. These cases indicate that through surface functionalization of membranes (such as the introduction of specific functional groups) and structural optimization, the targeted removal capability of HF-LNF membranes for specific OMPs can be significantly enhanced.

Some OMPs have molecular dimensions within the separation range reported for LNF membranes, suggesting that HF-LNF may be relevant for selected compounds. Nevertheless, molecular weight alone is insufficient to predict removal because OMP rejection also depends on charge state, hydrophobicity, adsorption, molecular shape, membrane charge, ionic strength, and feed-matrix effects. Direct HF-LNF evidence for broad-spectrum OMP removal remains limited. Based primarily on evidence from related LNF and HF membrane systems, potentially relevant research directions include prioritizing representative mixtures, using environmentally relevant concentrations and multicomponent feeds, conducting adsorption mass balances and transformation-product analyses, and performing extended filtration tests. These concepts remain to be validated in HF-LNF membranes under long-term module-scale operation. This will promote the further development of HF-LNF membrane technology in the field of emerging contaminant removal and provide more reliable technical support for safeguarding water environmental safety and human health.

The application studies discussed above primarily report initial permeance and solute rejection under selected laboratory conditions. However, short-term separation performance alone is insufficient to demonstrate the practical reliability of HF-LNF membranes. Long-term implementation also depends on the chemical stability of the selective layer, the interfacial stability between the selective layer and the HF substrate, and the mechanical integrity of the fibers and modules during prolonged filtration, chemical cleaning, and hydraulic loading. Table 6 therefore summarizes the currently available durability evidence for HF-LNF membranes and, where direct data are unavailable, supporting evidence from related HF-NF systems, including TFC and thin-film nanocomposite (TFN) configurations. Because the reported studies differ substantially in membrane configuration, exposure conditions, filtration mode, test duration, and performance indicators, the results should be interpreted as study-specific evidence rather than as a basis for direct quantitative comparison.

**Table 5:** List of the main OMP compounds and their properties.

Chemical Name (Abbreviation)	pK_a_	Molecular Weight (g/mol)	Density (g/cm^3^)	Spherical Diameter (nm)	Chemical Structure (3D)	Ref.
Atrazine (ATZ)	1.68	215.7	1.19	0.83	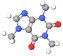	[207]
Benzophenone-1	7.7	182.2	1.11	0.8	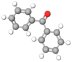	[208]
Bis(2-ethylhexyl) phthalate	N/A	390.5	0.99	1.08	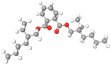	[209]
Bisphenol A (BPA)	9.6	228.3	1.2	0.84	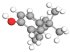	[210]
Butyl-paraben	8.5	194.2	1.1	0.82	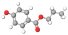	[211]
Chlorpyrifos (CPS)	4.5	350.6	1.4	0.93	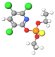	[212]
Ciprofloxacin	pKa1 = 3.01 pKa2 = 6.14 pKa3 = 8.70	331.3	1.5 ± 0.1	N/A	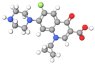	[205]
Perfluorooctane sulfonic acid (PFOS)	<1.0	500.1	0.8	1.26	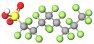	[213]
Perfluorooctanoic acid (PFOA)	2.8	414.1	1.8	0.9	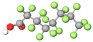	[214]
Tetracycline	pKa1 = 3.30, pKa2 = 7.70, pKa3 = 9.30	444	1.3809	N/A	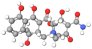	[204]

**Table 6:** Reported long-term operational, chemical, and mechanical durability of HF-LNF membranes and related hollow-fiber NF systems.

Membrane	Durability Test	Performance Response	Mechanical/Module	Ref.
SPSf-modified inner-selective HF-LNF	Continuous dye filtration, 76 h	Stable decolorization; quantitative retention NR	Cyclic pressure, burst strength, fiber-breakage data NR	[100]
Fiber-bundle-reinforced PES HF-LNF	Operation at 0.6 MPa, filtration, 100 h, cleaned 60 min after every 180 min	Flux: from 65.5 to 52.3 LMH dye rejection 99.9%, salt rejection < 7% FRR > 96%	Tensile strength: 185.7 MPa; little deformation	[215]
Fe–PA–PEI dual-coated HF-LNF	Filtration, 24 h, pH 3–11 exposure, three cleaning cycles	Normalized flux > 0.90 FRR: 93.2%	Maximum pressure withstood: 0.6 MPa	[175]
Polyelectrolyte-assisted polyurea HF-NF	10 wt.% H_2_SO_4_ 5 wt.% NaOH 28 days	Stable performance; In acidic conditions: permeance from 22.9 to 20.2 LMH/bar In alkaline conditions: permeance from 24.3 to 33.3 LMH/bar, R(MgCl_2_) from 93.3% to 89.0%	Fabricated as a 3/4-inch module; PWP from 24.4 to 21.8 LMH/bar, R(MgCl_2_) from 93.4% to 91.0%	[34]
Acid-resistant polyamine HF-NF	Continuous filtration with 0.1 M HCl, 15 d 2 bar	Stable flux: 20 LMH R(MgCl_2_): 94.8%	N/A	[216]
BPTAB-modified polyamine HF-NF	Immersion in 0.1 M HNO_3_, 60 d	Permeance: from 44.5 to 66.5 LMH/MPa R(MgCl_2_): from 97.3% to 92.9%	N/A	[217]
PEI–TDI polyurea HF-TFC	Extreme acid/alkali exposure, pH 1.5–13 45 d	MgCl_2_ rejection remained 91.6–93.9%	N/A	[218]
CuBTC- incorporated PA HF-NF TFN	1 bar TMP with 70 μg/L arsenic, pH 7, 36 h	Permeance: from 15.6 to 12.5 LMH R(arsenic): from 84.4% to 79.4%	N/A	[133]
GO-interlayered PA HF-NF TFN	0.5 MPa, 120 h; fouling evaluation	Permeance: 80 LMH/MPa; Na_2_SO_4_ rejection: 96.1%; FRR: 96.4	N/A	[131]

Note: HF-LNF indicates membranes classified as HF-LNF according to the operational definition adopted in this review. N/A: not reported; FRR: flux recovery ratio.

The available studies provide separate evidence of prolonged operation, resistance to selected chemical environments, and improved mechanical strength of hollow fibers. However, these properties are rarely evaluated simultaneously under module-relevant operating conditions. Prolonged acid or alkaline exposure alone does not fully represent the combined stresses encountered during continuous cross-flow filtration, which may include sustained or cyclic transmembrane pressure, periodic chemical cleaning, backwashing, and variations in hydraulic conditions. These stresses may lead to support compaction, selective-layer cracking, interfacial delamination, or loss of fiber and module integrity. Quantitative studies combining chlorine-cleaning cycles, cyclic pressure loading, selective-layer/substrate interfacial stability, and fiber or module integrity remain limited, particularly for HF-LNF systems. Standardized long-term cross-flow and module-scale durability tests are therefore required before different hollow-fiber membrane systems can be reliably compared.

## 5. Conclusions

HF-LNF integrates LNF selectivity with HF geometry and offers a potentially useful platform for separating organic solutes from saline water. Reported fabrication strategies, including phase inversion, IP, coating, and grafting, provide complementary routes for HF-LNF membrane development. Phase inversion offers process maturity and compatibility with continuous HF spinning but provides limited independent control over the support and selective structure. IP enables more precise regulation of selective-layer thickness, charge, and effective pore size, although defect control and interfacial durability remain important challenges. Coating and grafting are useful for tailoring hydrophilicity, charge, and antifouling properties, but their practical applicability depends on coating resistance, bonding stability, surface uniformity, and manufacturing reproducibility. Consequently, the preferred fabrication route should be selected according to the target separation and the required balance among performance, durability, process complexity, and scale-up feasibility. From an LCA perspective, current evidence is insufficient to show that HF-LNF is inherently more sustainable than alternative membrane configurations; its environmental performance depends on whether savings from compact modules and operation outweigh the burdens associated with membrane fabrication, cleaning, replacement, and end-of-life treatment.

Separation is governed by coupled steric, electrostatic, dielectric, hydration, adsorption, and concentration-polarization effects. Consequently, membrane performance cannot be interpreted reliably from MWCO or membrane charge alone. The available application studies demonstrate promising organic/salt fractionation, water-softening, heavy metal, and micropollutant-removal behavior under selected laboratory conditions. However, direct comparison is limited by differences in feed composition, pressure, pH, temperature, filtration mode, and test duration, and high initial rejection may partly reflect adsorption or solute aggregation.

Nevertheless, translating HF-LNF from laboratory research to industrial application still faces three major challenges: (1) the inherent trade-off between high flux, precise selectivity (especially for 200–500 Da neutral micropollutants), and long-term stability; (2) insufficient antifouling ability and separation accuracy in complex multicomponent wastewaters; and (3) dependence on multi-step, energy-intensive, or hazardous solvent-based manufacturing processes. To overcome these bottlenecks, future research should focus on (1) next-generation materials including self-healing polymers, COFs, MOFs, and bio-based building blocks, combined with novel transport mechanisms based on molecular recognition; (2) LCA-guided green manufacturing such as solvent-free IP and roll-to-roll production; and (3) scalable, low-cost processes including pressurized IP and renewable energy-driven systems.

Overall, HF-LNF represents a promising separation technology for sustainable water treatment. With continuous advances in material design, structural optimization, green manufacturing, and system engineering, next-generation HF-LNF membranes may play an increasingly important role in addressing global water scarcity, emerging contaminant control, and resource recovery in chemical and environmental engineering.

## Figures and Tables

**Table 1:** Proposed life-cycle stages, inventory items, and assessment indicators relevant to HF-LNF systems.

LCA Stage	Key Activities	Materials/Substances
Raw Material Acquisition	Collection of polymer materials Nanofillers Solvents Crosslinking agents and additives	Polymeric materials (PVDF, PES, PA, PSf, etc.) Nanofillers (CNT, GO, MoS_2_) Solvents (NMP, DMAc, DMF, DMSO) Crosslinking agents (MDI, epoxy compounds) Charge modifiers (carboxyl groups, sulfonic acid groups)
Membrane Manufacturing	Membrane fabrication (casting, polymerization, crosslinking) Solvent usage Energy consumption and waste generation	Energy consumption (electricity, thermal energy) Chemicals (solvents, catalysts, curing agents) Water consumption Wastes (spent solvents, plastics, chemicals)
Operational Phase	Operating conditions (pressure, temperature, flow rate) and energy consumption Cleaning and maintenance Fouling and flux decline	Operational energy Chemicals (cleaning agents, disinfectants) Water (feedwater, permeate) Contaminants (organic, inorganic substances)
End-of-Life Phase	Recycling potential Waste management Disposal methods (mechanical crushing, chemical degradation, incineration)	Wastes (plastics, metals, waste solvents) Membrane waste (landfill, recycling, incineration)
Environmental Impact Assessment	Carbon footprint evaluation Energy consumption Water consumption Waste generation and toxic emissions	Carbon footprint (CO_2_ emissions) Energy use (electricity, fuel) Water resource consumption Wastes (chemicals, plastics, etc.) Emissions (CO_2_, NO_x_, particulate matter)

Note: The listed items represent a proposed HF-LNF assessment framework rather than a complete inventory derived from HF-LNF case studies.

**Table 2:** The composition of dope solutions and spinning parameters of optimal HF membranes with different materials.

**Primary Material**	**Solvent**	Bore Liquid	Pore-Forming Agent	Co-Solvent	Air Gap (mm)	Dope Flow Rate	Bore Fluid Flow Rate	Take-Up Speed (m/min)	Ref.
PVDF	DMAc	DI water	LiCl	Tetrahydrofuran (THF)	900	N/A	N/A	1.8	[88]
Outer: PAI Inner: PES	NMP	Water	poly(ethylene glycol) LiCl	N/A	5	Outer: 2 g/min Inner: 6 g/min	8 mL/min	5.68	[90]
poly(m-phenylene isophthalamide) (PMIA)	DMAc	DI water	polyvinylpyrrolidone (PVP) LiCl	PVP	250	6 mL/min	3 mL/min	N/A	[38]
poly(vinyl chloride) (PVC)	NMP/THF (3:2)	DI water	PVP	THF	100	N/A	5 mL/min	Free fall	[91]
Polyetherimide (PEI)	DMAc	DI water	γ-butyrolactone (GBL)	N/A	40	N/A	N/A	15.1	[92]
polybenzimidazole (PBI)	NMP	Tap water	N/A	N/A	60	3 mL/min	2 mL/min	4.6	[93]
poly(phthalazinone ether sulfone) (PPES)	NMP/THF (8:2)	DI water	LiCl	diethylene glycol (DegOH)	50	1.5–2.0 mL/min	1.5–2.0 mL/min	N/A	[94]

**Table 3:** Comparison of membrane properties obtained by different types of monomers.

**Membrane**	Materials		Permeance (LMH/bar)	Application	Transmembrane Pressure (bar)	Feed Concentration	Feed Solute	Rejection (%)	Ref.
	**Aqueous Monomer**	Organic Monomer							
LNF	meso-erythritol (ME)	TMC	53.2	Dye desalination	4	Dye: 200 ppm Salt: 1000 ppm	Na_2_SO_4_ NaCl Congo red Direct red 23 Reactive blue2	11.0 5.6 99.6 95.2 99.6	[107]
NF	polyhexamethylene guanidine hydrochloride (PHGH)	TMC	17.2 1	Dye desalination	6	MgCl_2_: 0.01 mol/L	MgCl_2_	86.2	[125]
LNF	3,5-diaminobenzamide (3,5-DABA)	TMC	24.6	Dye desalination	1	Dye: 100 ppm Salt: 1000 ppm	Acid Fuchsin EBT MB CR MgCl_2_ NaCl	97.5 99.6 99.8 99.5 4.8 3.6	[126]
LNF	1,2-bis (N-aminoethyl imidazoline) ethane (BAIE)	1,3,5-tris(bromomethyl)benzene (TBB)	157.1	Dye desalination	N/A	CR: 100 ppm NaCl: 1000 ppm	CR NaCl	99.6 1.5	[108]
LNF	PIP and self-synthesized polyphenolic monomer (HCTT)	TMC	124.8	Dye desalination	2	Dye: 100 ppm Salt: 1000 ppm	Methyl violet MgSO_4_	95.0 5.4	[109]
LNF	water-soluble cyanocarbon nitride (WCN)	TMC	41.6	Organics/salts separation	4	Dye: 100 ppm Salt: 1000 ppm	MB Na_2_SO_4_ NaCl	99.9 31.5 11.4	[127]
HF-NF	PIP	TMC	12.6	lithium and magnesium separation	4	MgCl_2_ and LiCl mixture: 2000 ppm	MgCl_2_	94.6	[128]
HF-NF	1,4-bis(3-aminopropyl) piperazine (DAPP)	TMC	7.9		3	IMgCl_2_ and LiCl mixture: 2000 ppm	MgCl_2_ LiCl	73.4 23.5	[129]
NF	triethanolamine (TEOA)	TMC	11.5	Selective separation of divalent salt from monovalent salt	6	Salt: 5 mmol/L	Na_2_SO_4_ MgSO_4_ NaCl MgCl_2_	82.2 76.5 42.2 23	[130]
HF-NF	PIP and GO	TMC	8.39		N/A	Dye: 100 ppm Salt: 2000 ppm	Na_2_SO_4_	96.1	[131]
HF-NF	Chitosan and MPD	TMC	32.5 ± 0.5	Removal of arsenic from water	0.5	80 µg/L	Arsenate	74.7	[132]
HF-NF	MPD	CuBTC MOF and TMC	16.6 ± 0.5		0.5	70 µg/L	Arsenate	85.8	[133]
HF-RO	MPD	TMC	4.5	Desalination of brackish water	2	NaCl: 1000 ppm	NaCl	~99	[134]
HF-NF	PEI and PIP	TMC	18.2	Low-pressure water softening		MgCl_2_ and MgSO_4_: 1000 ppm	MgCl_2_ MgSO_4_	96.3 93.8	[135]

**Table 4:** Properties of commonly used dyes.

Dye	Molecular Weight (Da)	Charge	Molecular Structure
Congo red (CR)	696.6	negative	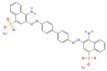
Methyl orange (MO)	327.3	negative	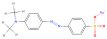
Direct yellow24 (DY24)	288.3	negative	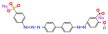
Acid orange10 (AO10)	452.3	negative	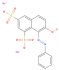
Rhodamine B (Rh.B)	479.0	N/A	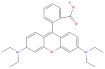
Neutral red (NR)	288.8	positive	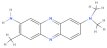
Crystal violet (CV)	407.9	positive	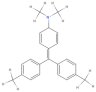

## Data Availability

No new data were created or analyzed in this study. Data sharing is not applicable to this article.
